# Applications of radiomics-based analysis pipeline for predicting epidermal growth factor receptor mutation status

**DOI:** 10.1186/s12938-022-01049-9

**Published:** 2023-02-21

**Authors:** Zefeng Liu, Tianyou Zhang, Liying Lin, Fenghua Long, Hongyu Guo, Li Han

**Affiliations:** 1 Department of Radiology, Tianjin Medical University General Hospital, Tianjin, 300052 People’s Republic of China; 2 First Central Clinical College, Tianjin Medical University, 22 Qixiangtai Road, Heping District, Tianjin, 300070 People’s Republic of China; 3 Department of Radiology, Institute of Hematology and Blood Diseases Hospital, Chinese Academy of Medical Sciences and Peking Union Medical College, Tianjin, 300041 People’s Republic of China; 4 School of Medical Imaging, Tianjin Medical University, 9-307, Guangdong Rd. #1, Hexi, Tianjin, 300203 People’s Republic of China; 5 Department of Radiology, University of Michigan, Ann Arbor, Michigan 48109 USA

**Keywords:** ^18^F-fluorodeoxyglucose positron emission tomography/computed tomography images, Radiomic, Epidermal growth factor receptor

## Abstract

**Background:**

This study aimed to develop a pipeline for selecting the best feature engineering-based radiomic path to predict epidermal growth factor receptor (EGFR) mutant lung adenocarcinoma in ^18^F-fluorodeoxyglucose (FDG) positron emission tomography/computed tomography (PET/CT).

**Methods:**

The study enrolled 115 lung adenocarcinoma patients with EGFR mutation status from June 2016 and September 2017. We extracted radiomics features by delineating regions-of-interest around the entire tumor in ^18^F-FDG PET/CT images. The feature engineering-based radiomic paths were built by combining various methods of data scaling, feature selection, and many methods for predictive model-building. Next, a pipeline was developed to select the best path.

**Results:**

In the paths from CT images, the highest accuracy was 0.907 (95% confidence interval [CI]: 0.849, 0.966), the highest area under curve (AUC) was 0.917 (95% CI: 0.853, 0.981), and the highest F1 score was 0.908 (95% CI: 0.842, 0.974). In the paths based on PET images, the highest accuracy was 0.913 (95% CI: 0.863, 0.963), the highest AUC was 0.960 (95% CI: 0.926, 0.995), and the highest F1 score was 0.878 (95% CI: 0.815, 0.941). Additionally, a novel evaluation metric was developed to evaluate the comprehensive level of the models. Some feature engineering-based radiomic paths obtained promising results.

**Conclusions:**

The pipeline is capable of selecting the best feature engineering-based radiomic path. Combining various feature engineering-based radiomic paths could compare their performances and identify paths built with the most appropriate methods to predict EGFR-mutant lung adenocarcinoma in ^18^FDG PET/CT. The pipeline proposed in this work can select the best feature engineering-based radiomic path.

**Supplementary Information:**

The online version contains supplementary material available at 10.1186/s12938-022-01049-9.

## Introduction

The development of computer hardware application in radiomics has facilitated progress in image analysis. Radiomics involves acquiring high-quality images, extracting and selecting features, analyzing results, and predictive model-building [1]. The technique allows for high-throughput and automatic extraction of numerous quantitative features from medical images, thus, aiding diagnosis. Radiomics is applicable for predicting many diseases [2–4], including the status of non-small cell lung cancer (NSCLC) [2, 5, 6].

Worldwide, lung cancer has the highest incidence and fatality rate [7, 8]. NSCLC is the typical type of lung cancer, with adenocarcinoma as the most common histological subtype [9]. Tyrosine kinase inhibitor (TKI) targeting epidermal growth factor receptor (EGFR) mutations significantly improves NSCLC prognosis in patients with EGFR mutations [10]. However, administering EGFR-TKI on NSCLC patients without EGFR mutations was ineffective and probably worsened prognosis than traditional treatment. Therefore, detecting EGFR mutation in NSCLC patient prognosis is crucial.

Mutation profiling of biopsies and surgically removed samples is the gold standard of EGFR mutation detection. However, the procedure is difficult and unjustified in clinical practice because of poor DNA quality, extensive heterogeneity of lung tumors, and difficulty accessing sufficient lung tissue [11, 12]. Therefore, radiomic technology is crucial for detecting non-invasive EGFR mutation.

Several recent studies have tested different new methods using large datasets [13, 14] or built clinical prediction models [15]. However, the models were built on a single path. Reasonable processing of radiomic features is essential for classifying NSCLC patients correctly. Feature engineering-based radiomic methods have different data scaling and feature selection methods and many methods for predictive model-building. However, these methods, when combined, result in many paths with different results. Therefore, selecting logical paths is significant for feature engineering-based radiomics.

This study built a pipeline of various data scaling, feature selection, and predictive model-building methods using ^18^F-fluorodeoxyglucose (FDG) positron emission tomography/computed tomography (PET/CT) images to select the best feature engineering-based radiomic path. Data scaling involved min–max algorithm, max-abs algorithm, and scale algorithm. Feature selection entailed variance threshold, Student's t-test, mutual information, embedded techniques, and least absolute shrinkage and selection operator (LASSO). The predictive models were built using logistic regression, decision tree, random forest, and support vector machine (SVM). Afterward, the accuracy, area under the curve (AUC), and F1 scores assessed the predictive power of the models. We proposed novel evaluation metrics, which is the weighted sum of the above three indicators, to evaluate the comprehensive level of the models.

## Results

### Radiomics features extraction

**Fig. 1:**
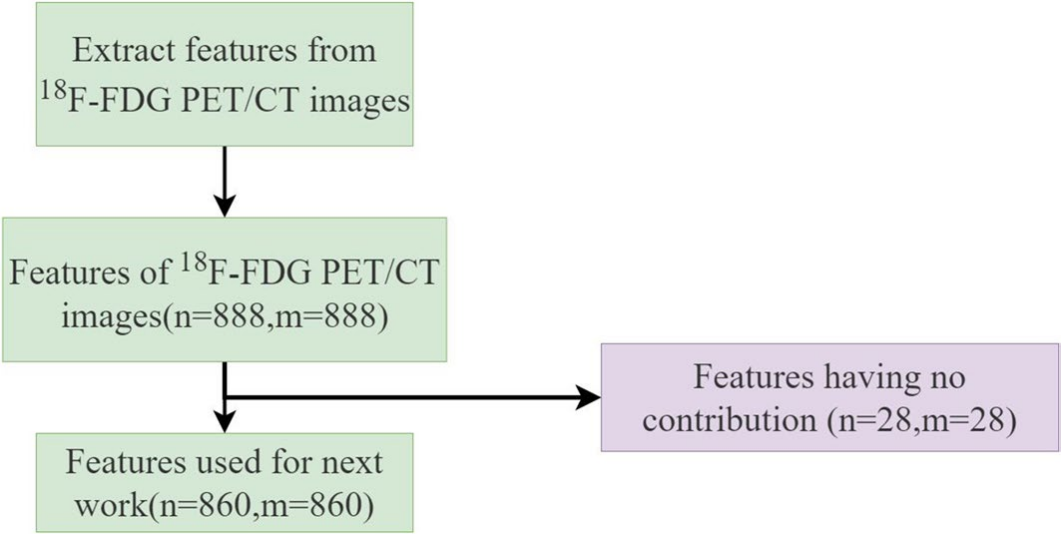
Flowchart of feature extraction and exclusion of ^18^F-FDG PET/CT images, where n and m are the numbers of features extracted from ^18^F-FDG PET/CT images

The study individually extracted 888 radiomics features each from CT and ^18^F-FDG PET. The study included 61 kinds of radiomics features. These features included original non-textural features (first-order statistics and shape-based) of images, textural features, and the textural features of wavelet-filtered and Gaussian-filtered images. Textural features included gray level co-occurrence matrix (GLCM) [16], gray level run length matrix (GLRLM) [17], gray level size zone matrix (GLSZM) [16], neighboring gray-tone difference matrix (NGTDM) [18], gray level dependence matrix (GLDM) [19]. Twenty-eight individual CT and 28 ^18^F-FDG PET image features that were duplicated or not contributing to later work were removed (Fig. 1).

### Feature selection

**Fig. 2:**
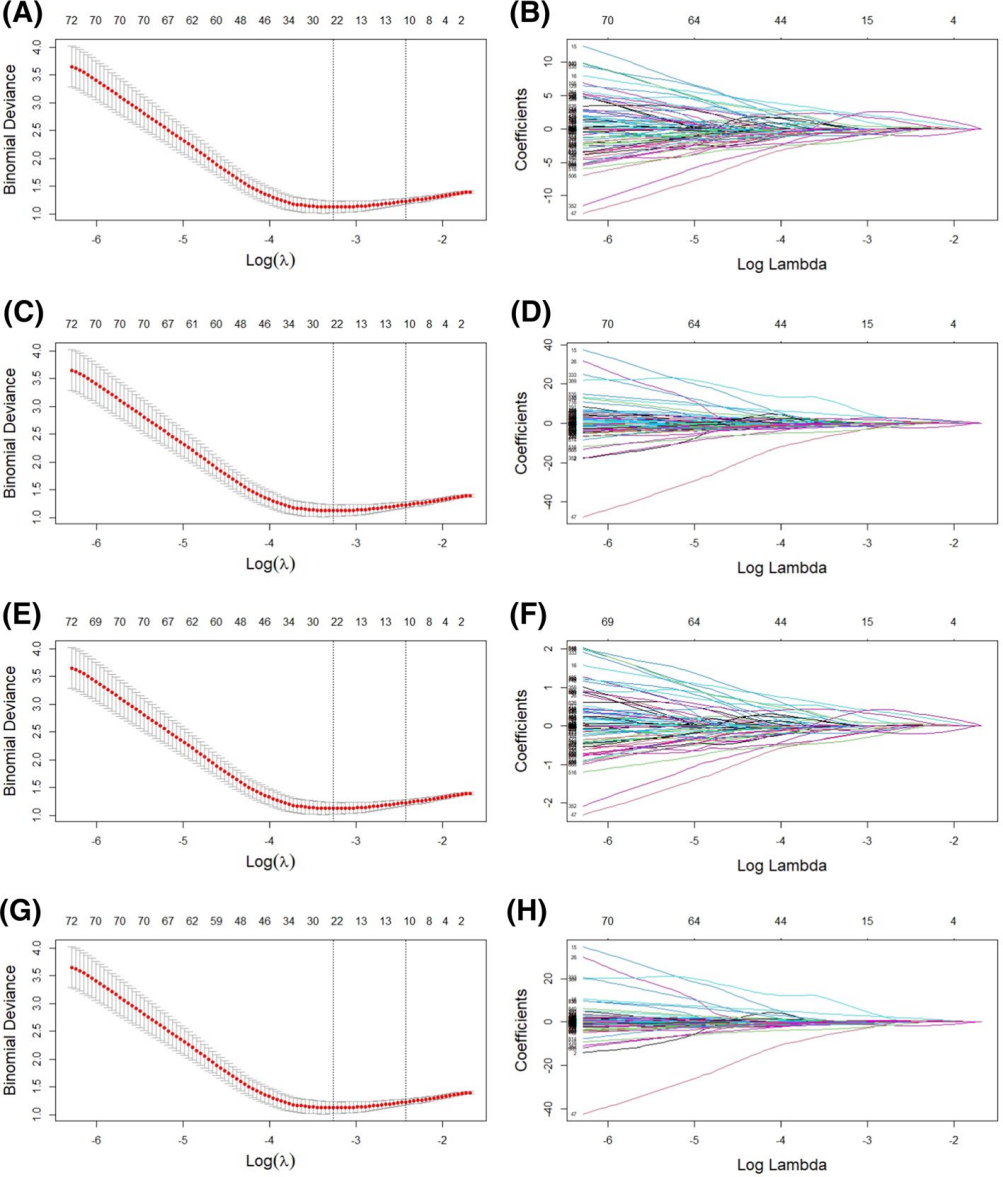
Radiomics features of CT image selected using the LASSO Cox regression model. **A**, **C**, **E**, and **G** Represent partial likelihood deviances drawn against the log (*λ*) of features after the min–max, max-abs, and Scale algorithm, and Scale algorithm without center-scaling. **B**, **D**, **F**, and **H** Represent the coefficients of selected features after the above algorithm scaling as shown by the lambda parameter

**Fig. 3:**
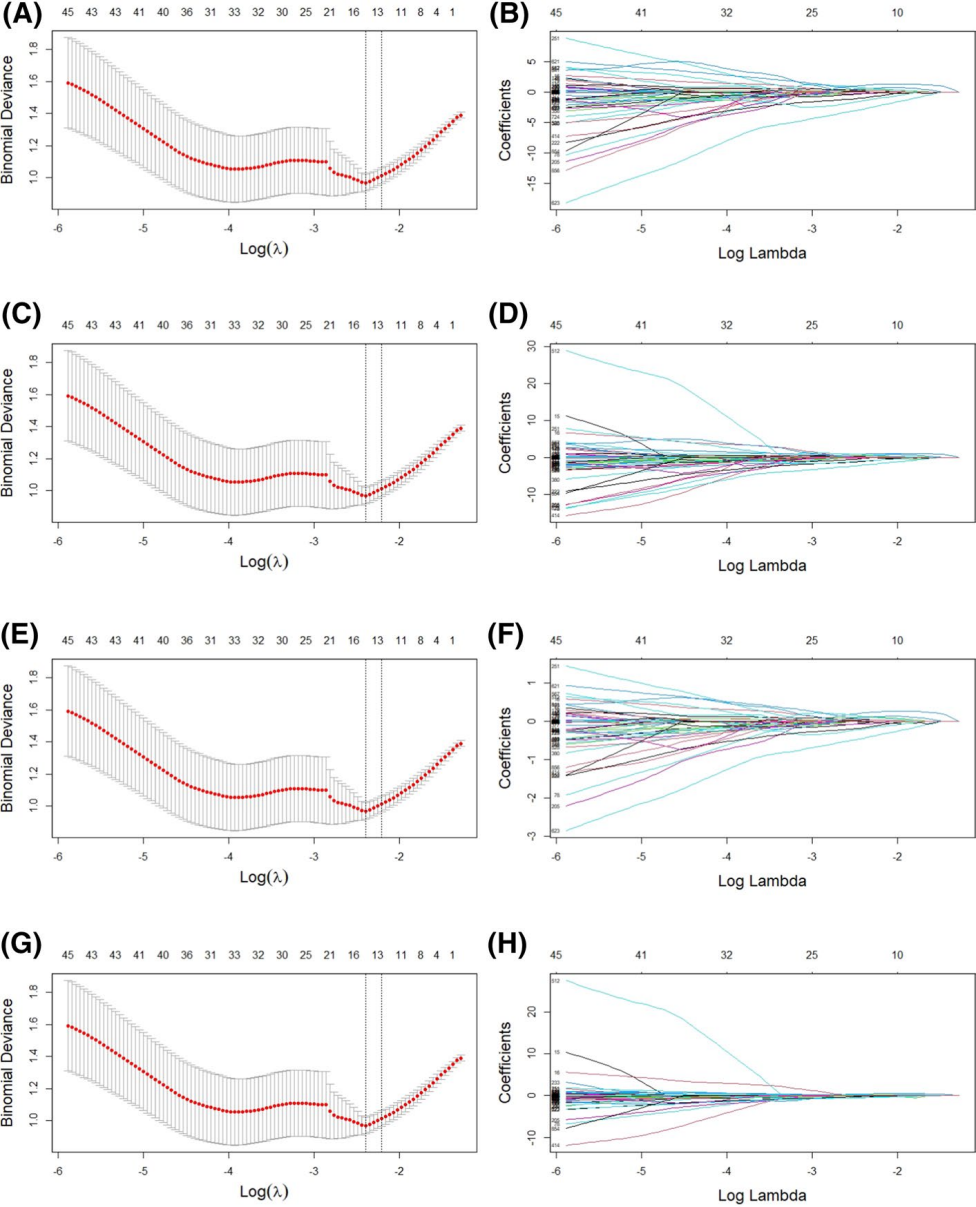
Radiomics features of PET images selected using the LASSO Cox regression model. **A**, **C**, **E**, **G** Represent partial likelihood deviances drawn versus log (*λ*) of features after min–max, max-abs, Scale algorithm, and Scale algorithm without center-scaling. **B**, **D**, **F**, **H** Represent the coefficients of selected features above algorithm scaling, shown by the lambda parameter

Variance threshold, *t*-test, mutual information, embedded solutions (the embedded capacity of logistic regression, decision tree, and random forest), and LASSO selected features for training the scaled data. Figures 2 and 3 show the results of feature selection using LASSO. The results of feature selection using other methods are shown in the supplement results (Additional file 2; see Additional files 3, 4). The number of remaining features after the above feature selection methods is presented in Tables 1 and 2.

**Table 1:** The number of remaining features after selection in CT images

Feature selection method	Data-scaling algorithm			
	Min–max algorithm	Max-abs algorithm	Scale algorithm	Scale algorithm without center-scaling
Variance threshold	393	393	393	393
T-test	184	183	185	184
Mutual information	517	537	538	538
Embedded capacity of logistic regression	205	75	137	81
Embedded capacity of decision tree	9	8	8	10
Embedded capacity of Random forest	50	15	50	50
LASSO	22	22	22	22

**Table 2:** The number of features remaining after selection in PET image

Feature selection method	Data scaling algorithm			
	Min–max algorithm	Max-abs algorithm	Scale algorithm	Scale algorithm without center -scaling
Variance threshold	375	375	375	375
t-test	508	509	510	508
Mutual information	660	657	660	653
Embedded capacity of logistic regression	18	34	28	18
Embedded capacity of decision tree	12	7	6	7
Embedded capacity of Random forest	17	17	21	21
LASSO	14	14	14	14

### Predictive model-building and predictive values

**Fig. 4:**
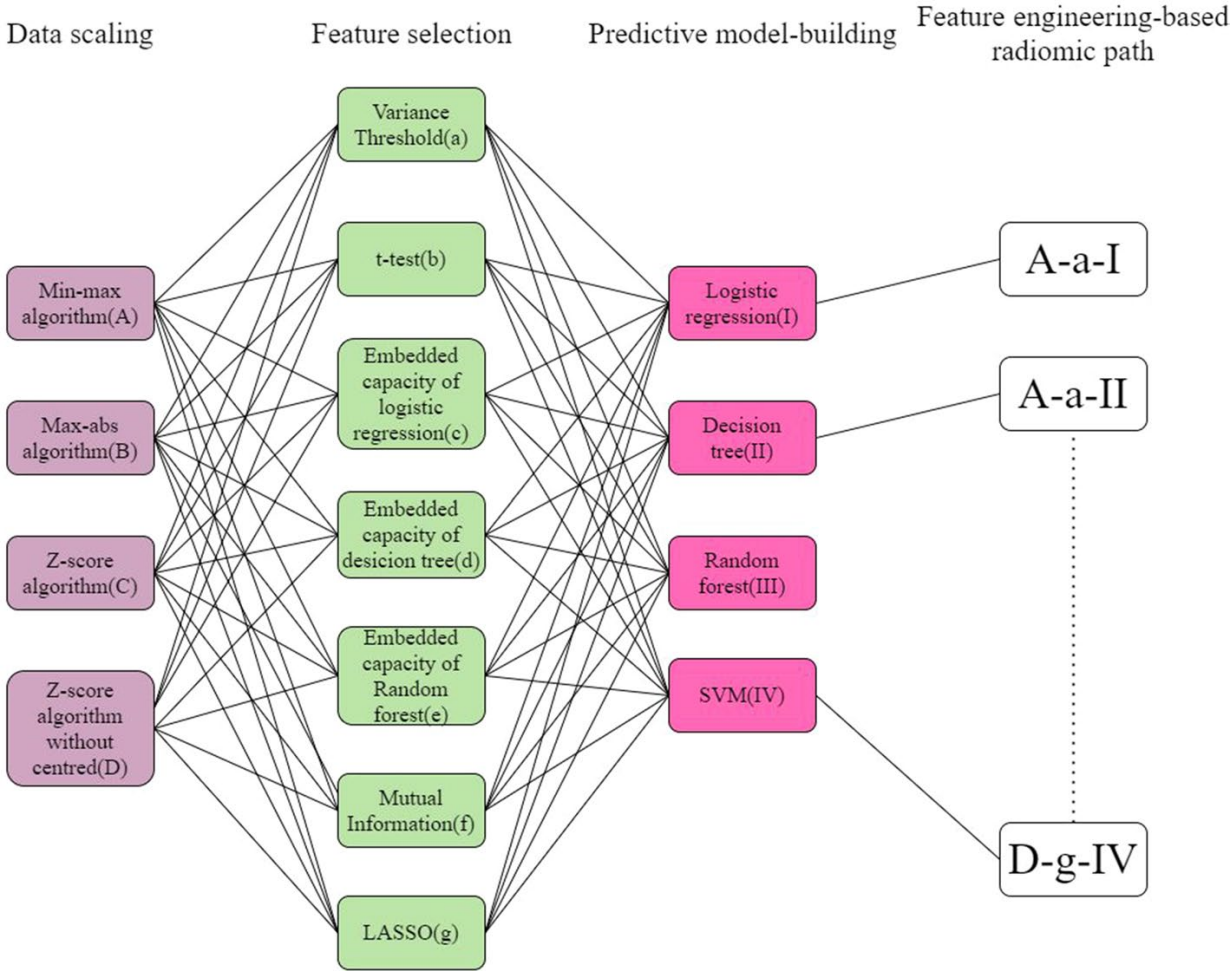
Feature engineering-based radiomic paths using different methods of data scaling, feature selection, and predictive model-building

**Fig. 5:**
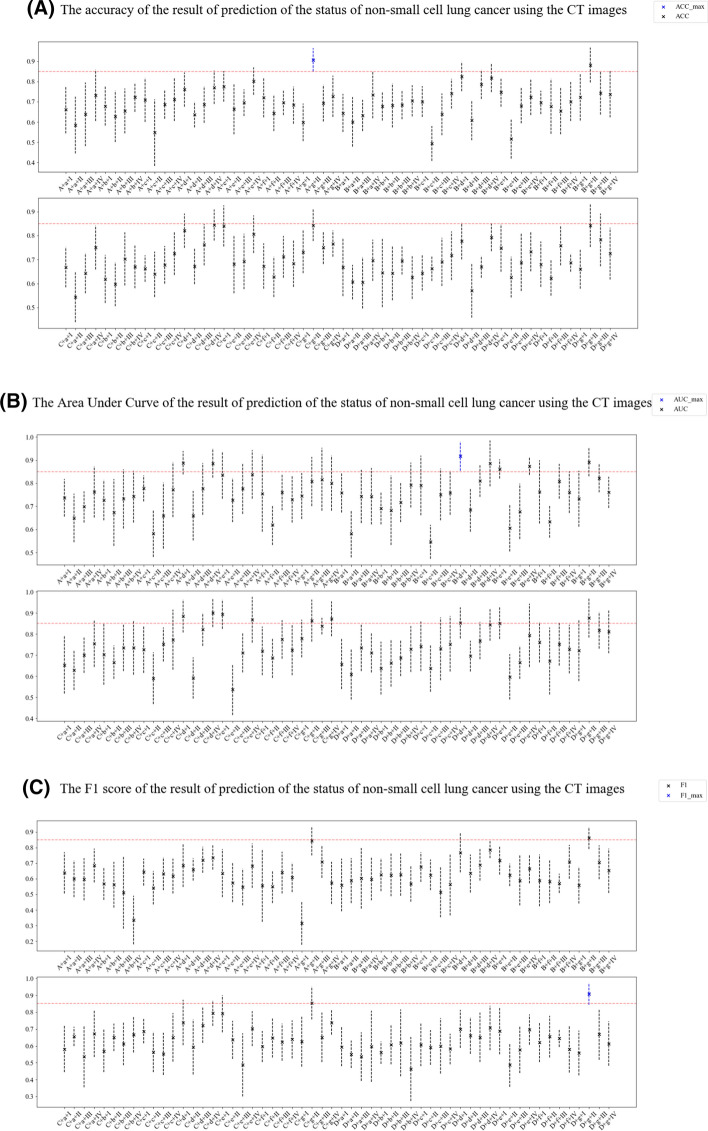

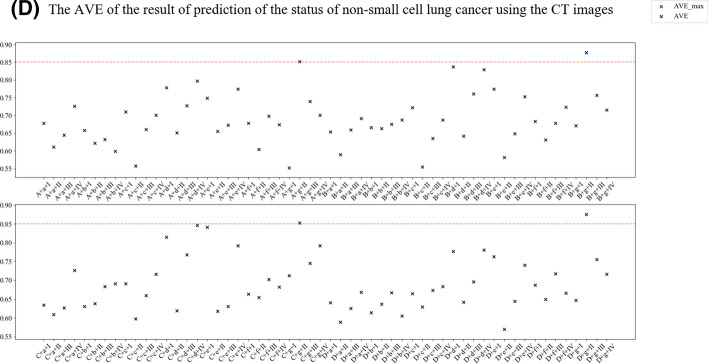
The accuracy (**A**), area under the curve (**B**), F1 scores (**C**) and AVE (**D**) of results predicting the status of non-small cell lung cancer using CT images

**Fig. 6:**
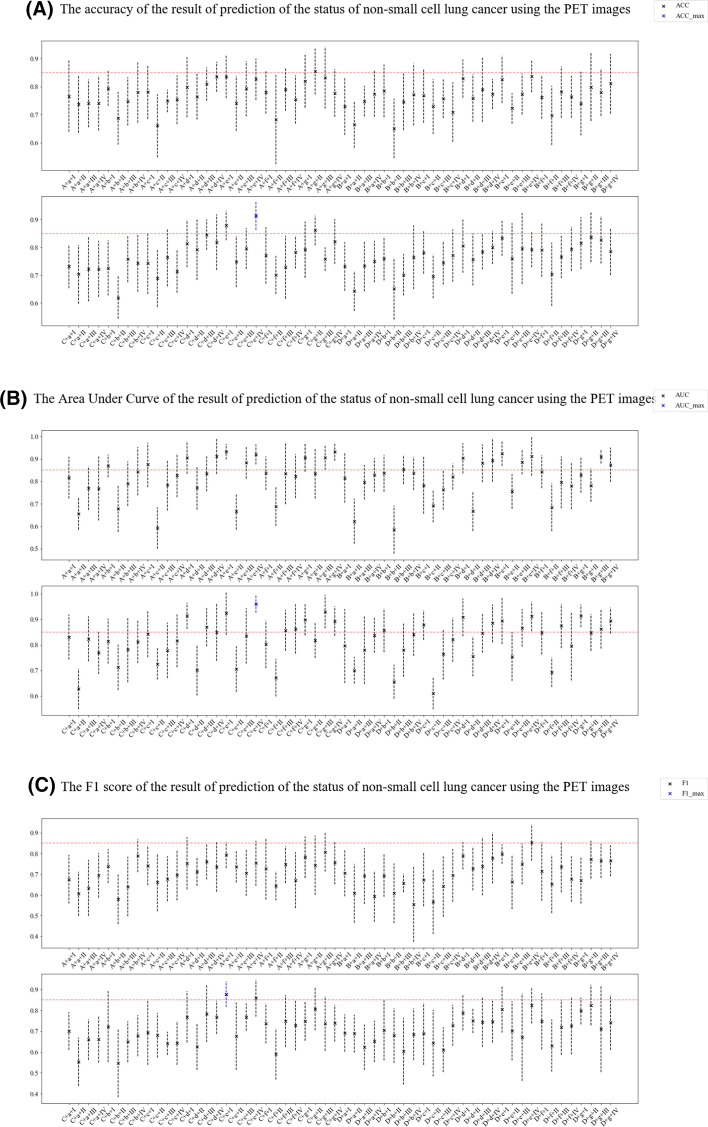

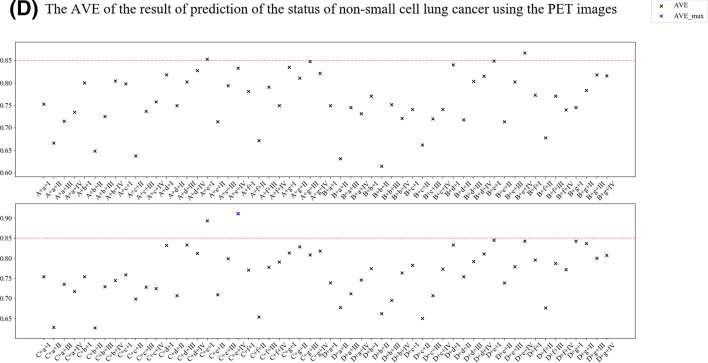
The accuracy (**A**), area under the curve (**B**), F1 scores (**C**) and AVE (**D**) of results predicting the status of non-small cell lung cancer using PET images

Tenfold cross-validation compared different feature engineering-based radiomic paths to predict the status of NSCLC using the ^18^F-FDG PET/CT images (Fig. 4). The accuracy, area under the curve (AUC), and F1 scores of the NSCLC prediction results from CT and ^18^F-FDG PET images (Figs. 5, 6). In order to reasonably select the effective models, the study proposed evaluation index (AVE). AVE is the average of above three indicators, which can evaluate the performance of various aspects of the model, as

1 $${\text{AVE = }}\frac{{({\text{ACC}} + {\text{ACU}} + {\text{F}}1{\text{score}})}}{3},$$

where *α*, *β*, and *γ* are defined as 1.00 in this study (Figs. 5 and 6). Table 3 shows the details of feature engineering-based radiomic paths with great prediction performances.

**Table 3:** The accuracy, area under the curve, F1 scores and AVE in feature engineering-based radiomic paths showing great prediction performances

Feature engineering-based radiomic path	ACC (95% CI)	AUC (95% CI)	F1 score (95% CI)	AVE
CT–A–g–II	0.907 (0.849, 0.966)	0.807 (0.701, 0.913)	0.842 (0.751, 0.934)	0.852
CT–B–d–I	0.826 (0.753, 0.898)	0.917 (0.853, 0.981)	0.767 (0.641, 0.893)	0.837
CT–D–g–II	0.843 (0.753, 0.932)	0.877 (0.784, 0.971)	0.908 (0.842, 0.974)	0.876
CT–B–g–II	0.881 (0.795, 0.967)	0.891 (0.829, 0.953)	0.862 (0.791, 0.933)	0.878
PET–C–e–IV	0.913 (0.863, 0.963)	0.960 (0.926, 0.995)	0.859 (0.770, 0.947)	0.911
PET–C–e–I	0.879 (0.825, 0.932)	0.924 (0.839, 1.000)	0.878 (0.815, 0.941)	0.894

*CI* confidence interval

In the paths whose radiomics features were extracted from CT images, the path CT–A–g–II obtained the highest ACC, CT–B–d–I obtained the highest AUC, and CT–D–g–II obtained the highest F1 score. Path CT–B–g–II obtained the highest AVE.

In the paths whose radiomics features were extracted from PET images, path PET–C–e–I obtained the highest F1 score. Path PET–C–e–IV obtained the highest ACC, AUC, and the AVE.

Different combinations of data-scaling algorithms, feature selection, and predictive models showed different performances in predicting the status of NSCLC. Predictive models from radiomics features of ^18^F-FDG PET images showed better prediction performance, but some radiomic paths from CT images showed greater prediction performance.

## Discussion

This study tried different feature engineering-based radiomic paths to predict the status of EGFR mutation for patients with lung adenocarcinoma. The study extracted radiomic features from CT and PET images of 115 patients for building predictive models. The data scaling involved the min–max, max-abs, Scale algorithm, and Scale algorithm without center-scaling. Moreover, feature selection used variance threshold, Student's *t*-test, mutual information, embedded techniques, and LASSO. The predictive model-building employed logistic regression, decision tree, random forest, and SVM. The results from comparing different feature paths revealed differences between these paths, with some paths showing excellent prediction performances (Table 3). These paths with excellent prediction performances will build models using small datasets and provide reference values for big training datasets.

Previous studies used different new methods and large datasets [13, 14] or built clinical prediction models to improve the performances of predictive models for EGFR mutation status [15]. For example, deep learning is used to predict the EGFR mutation status. This study built a pipeline trying different methods of data scaling, feature selection, and predictive model-building, and some paths showed good predictive ability. The study defined an index AVE to evaluate performance of the models in all aspects. The LASSO (g) and decision tree (II) achieved the greatest AVE indexes from CT images. The AVE of the CT–C–g–II path ranked third.

The paths that used Z-score (C) and embedded capacity of logistic regression (e) achieved significant indexes from PET images. The PET–C–e–I path obtained the highest F1 score. And although the PET–C–e–IV path does not obtained the highest F1 score, it obtained the highest AVE (Table 3). However, the five paths with the highest AVE included the embedded capacity of logistic regression (e) and logistic regression (I) or SVM (IV). Therefore, combining LASSO (g) and decision tree (II) can build the model for predicting the EGFR mutation status with excellent performance for CT images. However, the combination of the embedded capacity of logistic regression (e) and logistic regression (I) or embedded capacity of logistic regression (e) and SVM (IV) can build the predictive model for the EGFR mutation status with excellent performance using PET images.

The information in CT images and PET images is different, the CT images reflect the density and structure difference of tissue and the PET images reflect whether there are physiological or pathological changes in the human body at the molecular level. The different information leads to different the paths.

Choosing the best paths from the combination of standard methods in radiomic studies can better match the data than using different new methods. For some researchers, collecting a sufficient dataset is difficult. However, building a pipeline from different methods facilitates existing data to build a model with excellent performance. Researchers who have collected large datasets can build a pipeline to choose the best path, pre-train models with fewer data and use the minimum time to achieve an excellent training effect. The approach will positively influence future work on radiomics.

This study used AVE as an index to test the performance of models in all aspects. The CT–B–g–II path had the highest AVE, although the AUC, ACC, and F1 scores it achieved were not highest. The index defined in this study is not the most reasonable; thus, an index that can test the comprehensive level of the model is needed.

The study had several limitations. First, the CT and PET images used in this study are thick-slice. The thin-slice enhanced CT will be used to further improve the performance of models in subsequent work. Second, the tumor was manually segmented and potentially biased. The subsequent work will involve automatic or semi-automatic segmentation to improve experimental accuracy. Third, this study was single-centered, and the dataset had a relatively small sample size. Future work will use multi-centered datasets with large sample sizes. To an extent, these adjustments will increase the robustness of the models and make our views more persuasive.

## Conclusion

We built the pipeline system, trying many different methods of data scaling, feature selection, and many methods for predictive model-building in ^18^F-FDG PET/CT images to select the best feature engineering-based radiomic path for predicting the status of NSCLC. By analyzing the process of data scaling, feature selection, and predictive model-building, we established that some combinations could build the predictive models with excellent performance. The study also proved that many different combinations of methods could solve prediction problems. By trying many feature engineering-based radiomic paths, researchers will build predictive models with excellent performance.

## Materials and methods

### Ethical approval

The medical ethics committee of Tianjin Medical University Cancer Hospital approved this study, waived the necessity to obtain informed consent.

### Creation of dataset

This study collected the data of 550 patients who performed ^18^F-FDG PET/CT imaging before surgery or aspiration biopsy at Tianjin Medical University Cancer Hospital. The study recruited 152 patients with confirmed histopathological primary pulmonary adenocarcinoma. Patients included in this study met the following inclusion criteria:

1. Patients performed ^18^F-FDG PET/CT imaging before surgery or aspiration biopsy between June 2016 and September 2017.
2. The specimens obtained by surgical resection or aspiration biopsy were tested for EGFR mutation.
3. Patients had no tumor history.
4. The maximum tumor diameter was more than 1 cm.
5. Patients have not received neoadjuvant chemotherapy/radiotherapy before ^18^F-FDG PET/CT imaging.
6. The duration between surgery/biopsy and ^18^F-FDG PET/CT images was less than 2 weeks.

The exclusion criteria were:

1. Patients with low foci uptake that failed automatic delineation by the PETVCARr software (*n* = 27).
2. Multiple cavities were found in the tumor on PET/CT images (*n* = 10).

Finally, 115 patients (53 males and 62 females; mean age of 60.57 years ± 8.63; 51 EGFR-wild type, and 64 EGFR-mutant patients) were included in this study. The patient characteristics in datasets are shown in Table 4. This study followed the 1964 Helsinki declaration and later amendments or comparable ethical standards.

**Table 4:** Patient characteristics in datasets

Characteristic	Dataset (*N* = 115)		
	EGFR-wild type (*n* = 51)	EGFR-mutant type (*n* = 64)	*p* value
Age			0.425
Median	63	62.5	
Min	28	33	
Max	74	77	
Sex (*n, %*)			0.352
Male	26 (51.0)	27 (42.2)	
Female	25 (49.0)	37 (57.8)	
Smoking history (*n, %*)			0.042
Smoking or smoking in recent 5 years	21 (41.2)	15 (23.4)	
Never smoke	30 (58.8)	49 (76.6)	
TNM staging			0.810
I	33 (64.7)	46 (71.9)	
II	7 (13.7)	4 (6.2)	
III	10 (19.6)	11 (17.2)	
IV	1 (2.0)	3 (4.7)	

### ^18^F-FDG PET/CT examination, region-of-interest segmentation and radiomics feature extraction

This study obtained high-throughput quantitative NSCLC descriptors by delineating volume-of-interest (VOI) containing entire tumors, extracting and analyzing radiomics features of ^18^F-FDG CT images. The segmentation containing entire tumor in ^18^F-FDG PET and CT images was implemented using 3D Slicer (version 4.10.2) software. After 2 radiologists with 3- and 4-year experience in ^18^F-FDG PET/CT diagnosis performed the tumor segmentation in all patients, a 10-year experienced nuclear medicine physician confirmed their work.

Before extracting features, all images performed standardization to ensure the balance of the data. The supplementary information describes detailed ^18^F-FDG PET/CT procedure, parameters for CT image scanning, tumor region segmentation, and radiomics features extracted.

### Data scaling

Data scaling attempts to balance various datasets [20] and avoid different contributions to the data prediction in various numeric ranges.[21].

Four data-scaling algorithms, namely, min–max (A), max-abs algorithm (B), Z-score (C), and Z-score without center-scaling (D) algorithms compared this work.

#### Min–max algorithm (A)

The min–max algorithm linearly transformed the original data into [0, 1] intervals [22]. The Min–max algorithm mapped the original data D to data D’ as,

2 $$D^{\prime} = \frac{{D - D_{\min } }}{{D_{\max } - D_{\min } }},$$

where ${D}_{\mathrm{min}}$ and ${D}_{\mathrm{max}}$ represent the minimum and maximum values in the original data.

#### Max-abs algorithm (B)

The principle of max-abs algorithm is similar to the min–max algorithm. It scales the original data to [−1, 1] using linear mapping. The max-abs algorithm maps the original data *D* to data *D*’ as,

3 $$D^{\prime} = \frac{{D - D_{\mu } }}{{D_{\max } - D_{\mu } }},$$

where ${D}_{\mathrm{max}}$ and ${D}_{\mu }$ are the maximum value and average value in the original data.

#### Scale algorithm (C, D)

Scale algorithm is a function which can center and scale the original data D to data D’ as,

4 $$D^{\prime} = \frac{D - \mu }{\sigma },$$

where $\mu$ and $\sigma$ are the mean and standard deviations of the variables in the original data [22, 23]. After scaling, the treated data are normally distributed. This algorithm can also just scale the original data without center, as

5 $$D^{\prime} = \frac{D}{\sigma }.$$

In what follows, we use C to describe the Scale algorithm which can center and scale the original data, and use D to describe the Scale algorithm which can just scale.

### Feature selection

Feature selection obtains a subset of features, following specific feature selection criteria from an original feature set [24]. Feature selection processes high-dimensional data and enhances learning efficiency [25, 26] with other proven advantages [24, 27–29].

This work compared the effect of variance threshold (a), Student's *t*-test (*t*-test) (b), mutual information (c), embedded techniques (embedded capacity of logistic (d), embedded capacity of random forest (e), embedded capacity of decision tree (f)), and LASSO (g).

#### Variance threshold (a)

The mission of the variance threshold is to remove the features affecting the prediction little. The variance threshold considered features with a variance threshold of 3, thus, removing features whose variances do not meet the threshold.

#### Student's *t*-test (b)

The Student's *t*-test assumes that the null hypothesis is true and can test statistics following a Student’s *t*-distribution [30]. For a binary outcome, when the value of a continuous input variable for one population is significantly different from the other population, both populations are considered independent. Therefore, a t-test selects dependent features by retaining a two-sided *p* < 0.05 [29, 31].

#### Embedded techniques (c, d, e)

Embedded techniques use the classifier to search an optimal subset of features [32]. The technique removes features with minimal weights in classifiers. The technique also embeds in many different classifiers, including logistic regression [33], decision tree, random forest [34, 35], and SVM [36, 37]. This study employed the embedded capacity of logistic regression (c), decision tree (d), and random forest (e) to select the features.

#### Mutual information (f)

Mutual information measures the shared information between two variables, reflecting the dependence between two random variables [38, 39] at [0, 1] range. The mutual information is zero when the two random variables are independent and one when the variables are related. Therefore, mutual information is used for feature selection [39].

#### LASSO (g)

Tibshirani et al. [40] proposed LASSO for selecting features for linear-regression models compression from recent studies [3, 41–43]. The LASSO penalty term generated a regression model [44] whose outputs can fit classification label by employing the L1 norm for penalizing. Features with zero nonsignificant regressor coefficients were removed from the model [45–47].

### Predictive model-building

After data scaling and feature selection, feature selection models were built to predict the status of NSCLC. There are many methods for predictive model-building, including machine-learning methods. Recent studies have used machine-learning methods to predict the NSCLC status [2, 5, 6]. Machine-learning is a subfield of artificial intelligence where computers learn from available complex data [48, 49]. This work compared four machine-learning methods, including logistic regression (I), decision tree (II), random forest (III), and SVM (IV).

#### Logistic regression (I)

This machine-learning method analyzes the relationship between multiple independent variables and one categorical dependent variable [50, 51]. Logistic regression is usually used for binary classification, and in recent years, radiomics [52]. In this study, the penalty and solver of Logistic regression were L2 regularization and liblinear, respectively.

#### Decision tree (II)

The decision tree is a regression model [53] produced by learning simple decision rules repeatedly and stacking these rules together without parameters. The model is a relatively straightforward method to learn a tree from such data [54]. In this study, the max depth of decision tree were 100.

#### Random forest (III)

Random forest is a bagging ensemble approach based on decision trees [55] where decision trees are the “weak learners” in ensemble terms [56]. Random forest follows the majority rule, where the minority is subordinate to the majority. This approach considers the most common result of decision trees as the last result. In this study, Random forest had 10 decision trees.

#### SVM (IV)

SVM is also a widely used, supervised learning model for classification [57]. The SVM model classifies two classes with an optimal hyperplane that can separate all objects of both classes while keeping the largest margins between them. This study applied the kernel function of SVM as the radial basis function (RBF).The penalty term of SVM and kernel of RBF are optimized by cross-validated grid-search over a parameter grid.

Finally, this study proposed the above-mentioned methods of data scaling, feature selection, and predictive model-building of ^18^F-FDG PET/CT images to select the best feature engineering-based radiomic path.

## Supplementary Information

**Additional file 1.** Supplementary Information.

**Additional file 2.** Additional Results.

**Additional file 3.** Results of radiomic feature selection in CT images.

**Additional file 4.** Results of radiomic feature selection in PET images.

**Additional file 5.** Results of radiomic feature selection by Variance threshold in CT images.

**Additional file 6.** Results of radiomic feature selection by Variance threshold in PET images.

**Additional file 7.** Results of radiomic feature selection by t-test and Min-max algorithm in CT images.

**Additional file 8.** Results of radiomic feature selection by t-test and Max-abs algorithm in CT images.

**Additional file 9.** Results of radiomic feature selection by t-test and Scale algorithm in CT images

**Additional file 10.** Results of radiomic feature selection by t-test and Scale algorithm without center -scaling in CT images.

**Additional file 11.** Results of radiomic feature selection by t-test and Min-max algorithm in PET images.

**Additional file 12.** Results of radiomic feature selection by t-test and Max-abs algorithm in PET images.

**Additional file 13.** Results of radiomic feature selection by t-test and Scale algorithm in PET images.

**Additional file 14.** Results of radiomic feature selection by t-test and Scale algorithm without center -scaling in PET images.

**Additional file 15.** Results of radiomic feature selection by mutual information and Min-max algorithm in CT images.

**Additional file 16.** Results of radiomic feature selection by mutual information and Max-abs algorithm in CT images.

**Additional file 17.** Results of radiomic feature selection by mutual information and Scale algorithm in CT images.

**Additional file 18.** Results of radiomic feature selection by mutual information and Scale algorithm without center -scaling in CT images.

**Additional file 19.** Results of radiomic feature selection by mutual information and Min-max algorithm in PET images.

**Additional file 20.** Results of radiomic feature selection by mutual information and Max-abs algorithm in PET images.

**Additional file 21.** Results of radiomic feature selection by mutual information and Scale algorithm in PET images.

**Additional file 22.** Results of radiomic feature selection by mutual information and Scale algorithm without center -scaling in PET images.

## Data Availability

The datasets used and/or analyzed during the current study are available from the corresponding author on reasonable request.

## Abbreviations

EGFR: Epidermal growth factor receptor
CI: Confidence interval
ACC: Accuracy
NSCLC: Non-small cell lung cancer
TKI: Tyrosine kinase inhibitor
LASSO: Least absolute shrinkage and selection operator
SVM: Support vector machine
GLCM: Gray level co-occurrence matrix
GLRLM: Gray level run length matrix
GLSZM: Gray level size zone matrix
NGTDM: Neighboring gray-tone difference matrix
GLDM: Gray level dependence matrix
