# Correction: Applications of radiomics‑based analysis pipeline for predicting epidermal growth factor receptor mutation status

**DOI:** 10.1186/s12938-023-01095-x

**Published:** 2023-03-29

**Authors:** Zefeng Liu, Tianyou Zhang, Liying Lin, Fenghua Long, Hongyu Guo, Li Han

**Affiliations:** 1 Department of Radiology, Tianjin Medical University General Hospital, Tianjin, 300052 People’s Republic of China; 2 First Central Clinical College, Tianjin Medical University, 22 Qixiangtai Road, Heping District, Tianjin, 300070 People’s Republic of China; 3 Department of Radiology, Institute of Hematology and Blood Diseases Hospital, Chinese Academy of Medical Sciences and Peking Union Medical College, Tianjin, 300041 People’s Republic of China; 4 School of Medical Imaging, Tianjin Medical University, 9‑307, Guangdong Rd. #1, Hexi, Tianjin, 300203 People’s Republic of China; 5 Department of Radiology, University of Michigan, Ann Arbor, Michigan 48109 USA

**Correction** **:** **BioMedical Engineering OnLine (2023) 22:17** **https://doi.org/10.1186/s12938-022-01049-9**

Following publication of the original article [1], the affiliation for the author’s Tianyou Zhang, Liying Lin, Fenghua Long, Hongyu Guo has been updated and shown below:

Zefeng Liu^1^, Tianyou Zhang^2^, Liying Lin^3^, Fenghua Long^4^, Hongyu Guo^4*^ and Li Han^4,5*^

^1^Department of Radiology, Tianjin Medical University General Hospital, Tianjin 300052, People’s Republic of China

^2^First Central Clinical College, Tianjin Medical University, 22 Qixiangtai Road, Heping District, Tianjin 300070, People’s Republic of China

^3^Department of Radiology, Institute of Hematology and Blood Diseases Hospital, Chinese Academy of Medical Sciences and Peking Union Medical College, Tianjin 300041, People’s Republic of China

^4^School of Medical Imaging, Tianjin Medical University, 9‑307, Guangdong Rd. #1, Hexi, Tianjin 300203, People’s Republic of China

^5^Department of Radiology, University of Michigan, Ann Arbor, Michigan 48109, USA

The original article has been corrected.
