# Supplementary material for: Applications of radiomics-based analysis pipeline for predicting epidermal growth factor receptor mutation status
Source: Biomed Eng Online. 2023 Feb 21;22:17. doi: 10.1186/s12938-022-01049-9 (PMC9945395; doi:10.1186/s12938-022-01049-9)
Supplement: Supplementary file 1 — Additional file 1. Supplementary Information. [file 12938_2022_1049_MOESM1_ESM.docx]

Addition

2.2.^18^F-FDG PET/CT examination, region-of-interest segmentation and radiomics feature extraction

All baseline images retrieved from the picture archive and communication system database were analyzed using one scanner (GE Discovery Elite PET/CT scanner, GE Medical Systems,).Before taking PET/ CT image, all patients were fasted for about 6h and their serum glucose level <11.1mmol/L.Images were acquired 50-60 minutes after injection of 4.2 MBq/kg ^18^F-FDG.

In precise anatomical localization and attenuation correction,the spiral CT scanning parameters used in this study were as follows:tube voltage, 120 kV; 80 mAs; pitch, 0.75; rotation time, 0.6 s; slice thickness, 5 mm.

Then PET emission scaned the distal femur to the top of the skull. It take 2 min to scan per bed position .The increments is 16.2 cm (3D mode).All of the patients were scanned in eight bed positions. Used iterative algorithms to reconstruct the PET images. After the reconstruction, used a 6-mm full-width at half maximum Gaussian filter to perform filtering.

The segmentation containing entire tumor in ^18^F-FDG PET and CT images was implemented using 3D Slicer (version 4.10.2) software.After 2 radiologists with 3 and 4-year experience in ^18^F-FDG PET/CT diagnosis performed the tumor segmentation in all patients, a 10-year experienced nuclear medicine physician confirmed their work.

The features extracted by “PyRadiomics” (<http://www.radiomics.io/pyradiomics.html>) software (version 1.3.0). We extracted 888 radiomics features from CT images and 888 radiomics features from ^18^F-FDG PET images individually. 61 kinds of radiomics features are included. These features include original images’ non-textural features (First Order Statistics and Shape-based) and textural features, as well as the textural features of wavelet filtered images and gaussian filtered images. Textural features include Gray Level Co-occurrence Matrix(GLCM)(1), Gray Level Run Length Matrix (GLRLM)(2), Gray Level Size Zone Matrix(GLSZM)(1), Neighboring Gray Tone Difference Matrix(NGTDM)(3), Gray Level Dependence Matrix(GLDM)(4). Because some features are same or have no contribution to later work, we remove 28 CT images’ features and 28 ^18^F-FDG PET images’ features individually.

**References**

1. Thibault G, Fertil B, Navarro C, Pereira S, Cau P, Levy N, et al. Shape and texture indexes application to cell nuclei classification. International Journal of Pattern Recognition and Artificial Intelligence. 2013;27(01):1357002.

2. Galloway MM. Texture analysis using grey level run lengths. NASA STI/Recon Technical Report N. 1974;75:18555.

3. Amadasun M, King R. Textural features corresponding to textural properties. IEEE Trans Syst Man Cybern. 1989;19(5):1264-74.

4. Thibault G, Angulo J, Meyer F. Advanced statistical matrices for texture characterization: application to cell classification. IEEE Trans Biomed Eng. 2013;61(3):630-7.

**The results of feature selection**

The radiomics features selected results are shown in the CSV files named ‘CT_feature selection result.csv’ and ‘PET_feature selection result.csv’ .In the tables,0 means the features are removed and 1 means the features are selected.

**The results of Variance threshold**

The radiomics features of CT images selected results using Variance threshold are shown in the CSV file named ‘CT_Variance threshold.csv’.The radiomics features of PET images selected results using t-test are shown in the CSV files named ‘PET_Variance threshold.csv’.

In these files the rows named ‘Variance’ mean the variance of radiomics features. In the last rows , 0 means the features are removed and 1 means the features are selected.

**The results of t-test**

The radiomics features of CT images selected results using t-test are shown in the CSV files named ‘CT_minmax_t-test.csv’, ‘CT_maxabs_t-test.csv’, ‘CT_z-score_t-test.csv’ and ‘CT_z-score-no-center_t-test.csv’.The radiomics features of PET images selected results using t-test are shown in the CSV files named ‘PET_maxabs_t-test.csv’, ‘PET_minmax_t-test.csv’, ‘PET_z-score_t-test.csv’ and ‘PET_z-score-no-center_t-test.csv’.

In these files the rows named ‘levene’ mean the results of Levene test of radiomics features. The rows named ‘ttest’ mean the results of t-test test of radiomics features. In the last rows , 0 means the features are removed and 1 means the features are selected.

**The results of Mutual information**

The radiomics features of CT images selected results using t-test are shown in the CSV files named ‘CT_minmax_fsmic’, ‘CT_maxabs_fsmic.csv’, ‘CT_z-score_fsmic.csv’ and ‘CT_z-score-no-center_fsmic.csv’. The radiomics features of PET images selected results using t-test are shown in the CSV files named ‘PET_minmax_fsmic’, ‘PET_maxabs_fsmic.csv’, ‘PET_z-score_fsmic.csv’ and ‘PET_z-score-no-center_fsmic.csv’.

In these files the rows named ‘mutual information’ mean the results of mutual information of Radiomics features. In the last rows , 0 means the features are removed and 1 means the features are selected.

**The results of embedded solutions**

**The results of the embedded capacity of logistic regression**

**
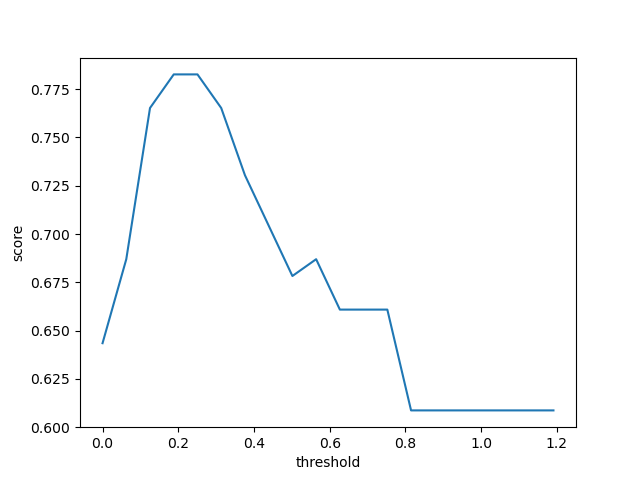
**

(A)

**
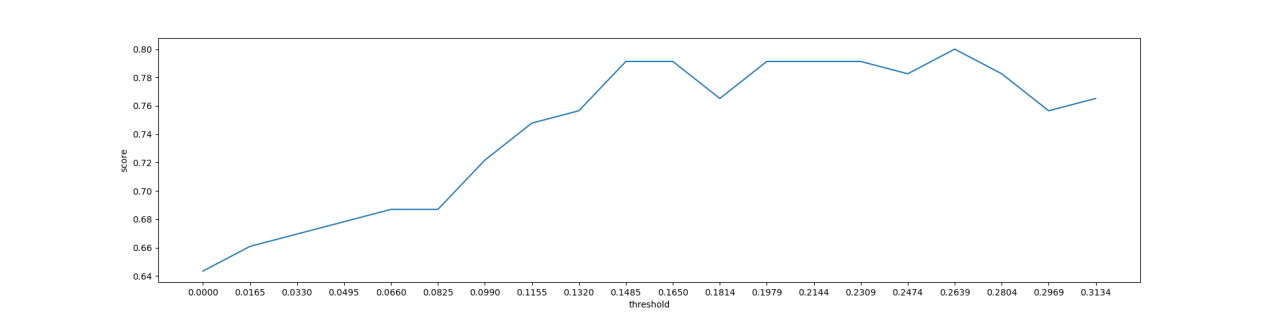
**

(B)

Fig.1 Radiomics features of CT image scaled using min-max algorithm and selected using the embedded capacity of logistic regression.(A) represent the relationship between the threshold of feature selection and the logistic regression model’s built by the features selected. (B) is the details of them.


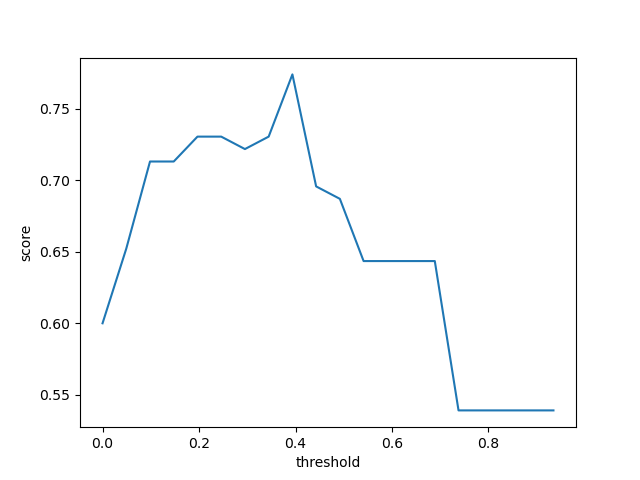


(A)


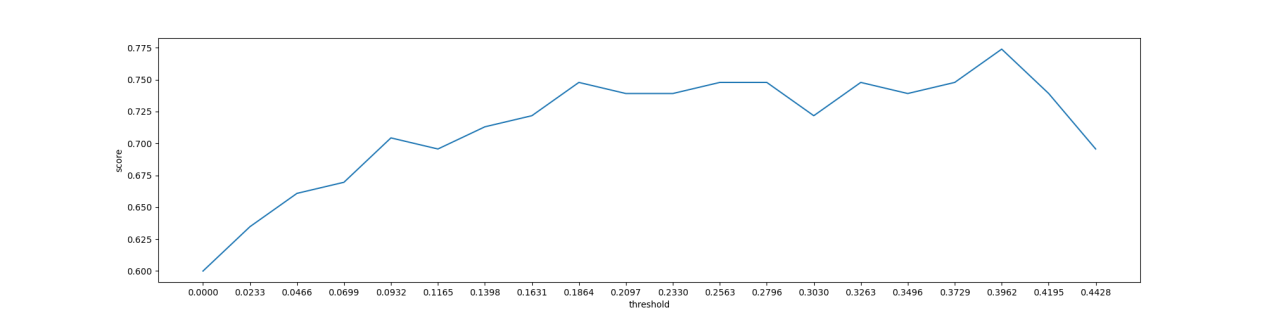


(B)

Fig.2 Radiomics features of CT image scaled using max-abs algorithm and selected using the embedded capacity of logistic regression.(A) represent the relationship between the threshold of feature selection and the logistic regression model’s built by the features selected. (B) is the details of them.


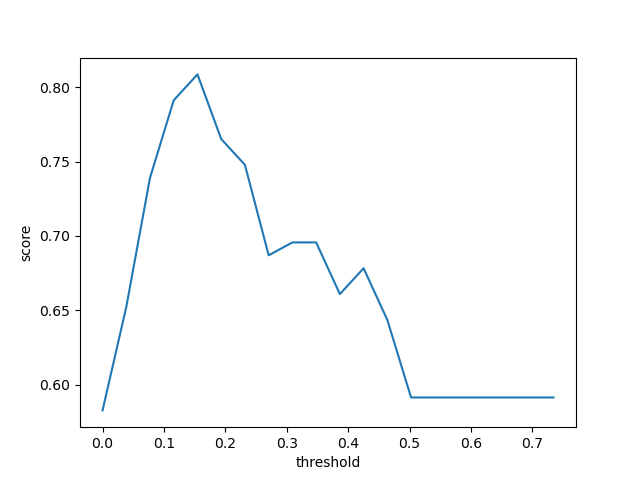


(A)


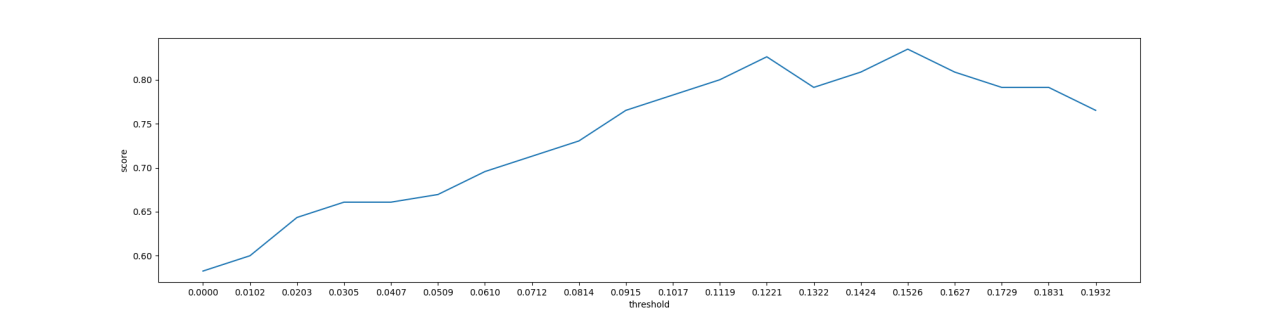


(B)

Fig.3 Radiomics features of CT image scaled using Scale algorithm and selected using the embedded capacity of logistic regression.(A) represent the relationship between the threshold of feature selection and the logistic regression model’s built by the features selected. (B) is the details of them.


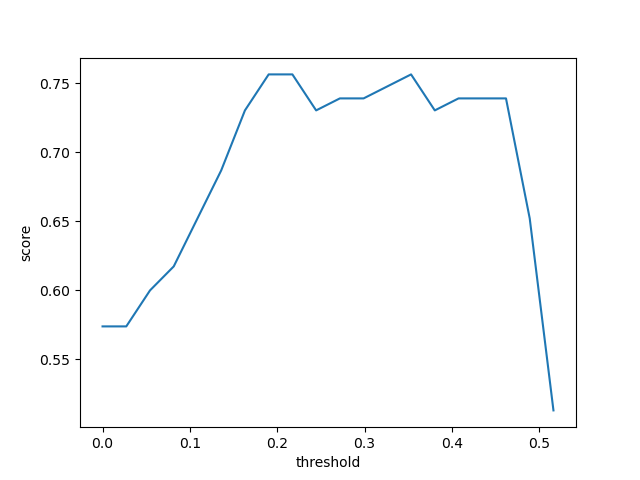


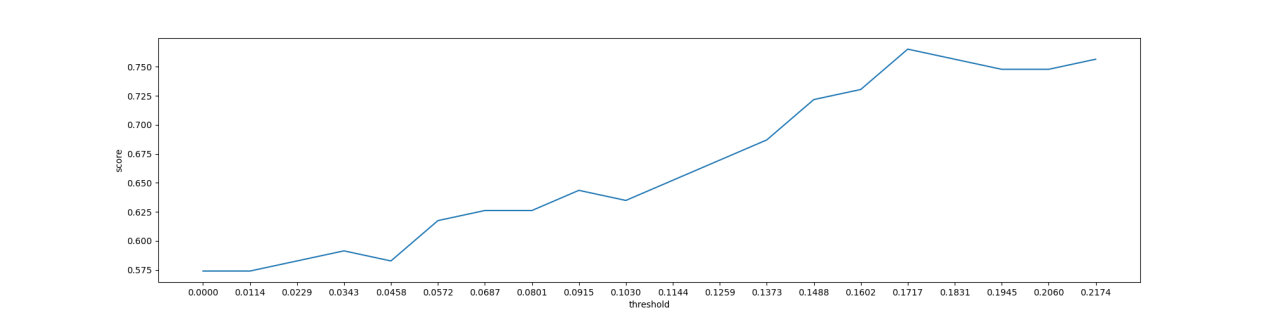


Fig.4 Radiomics features of CT image scaled using Scale algorithm without center -scaling and selected using the embedded capacity of logistic regression.(A) represent the relationship between the threshold of feature selection and the logistic regression model’s built by the features selected. (B) is the details of them.


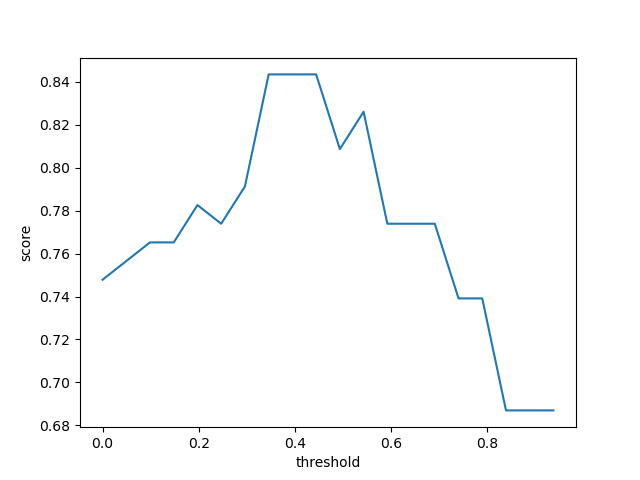


(A)


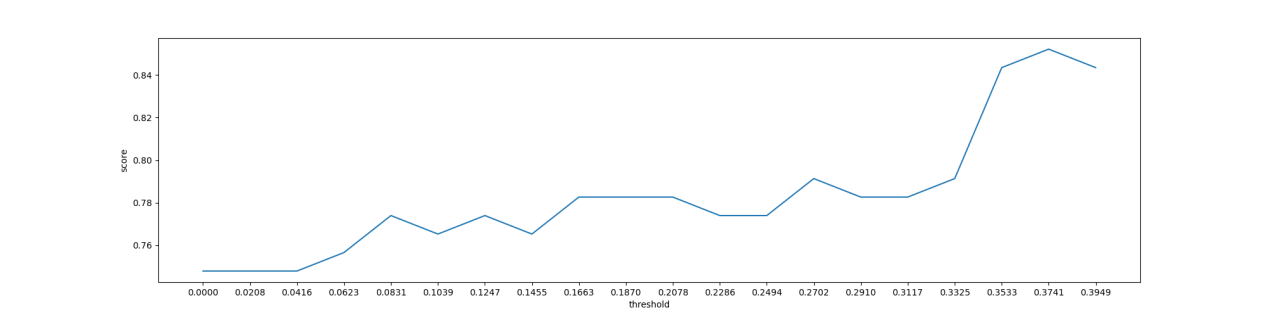


(B)

Fig.5 Radiomics features of PET image scaled using min-max algorithm and selected using the embedded capacity of logistic regression.(A) represent the relationship between the threshold of feature selection and the logistic regression model’s built by the features selected. (B) is the details of them.


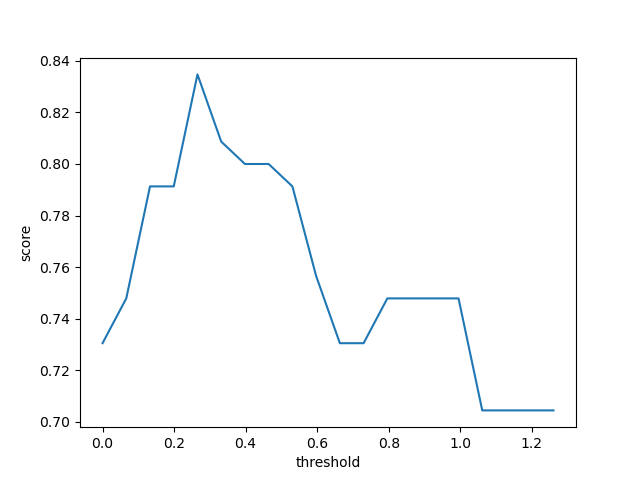


(A)


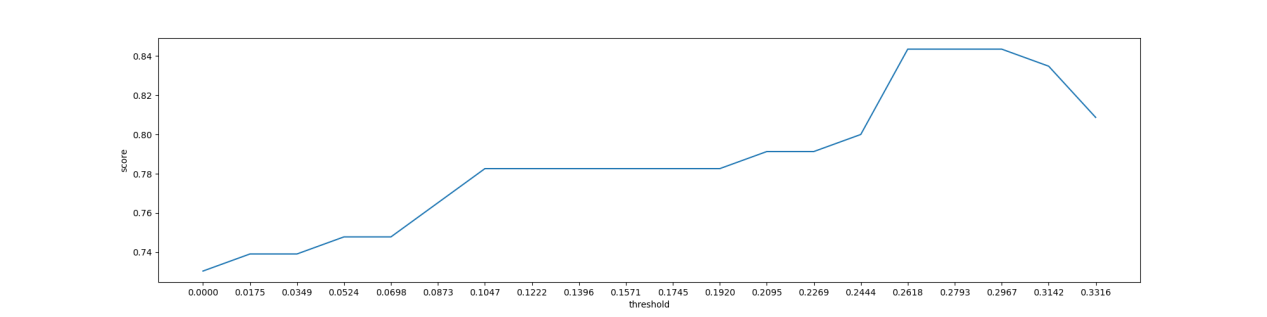


(B)

Fig.6 Radiomics features of PET image scaled using max-abs algorithm and selected using the embedded capacity of logistic regression.(A) represent the relationship between the threshold of feature selection and the logistic regression model’s built by the features selected. (B) is the details of them.


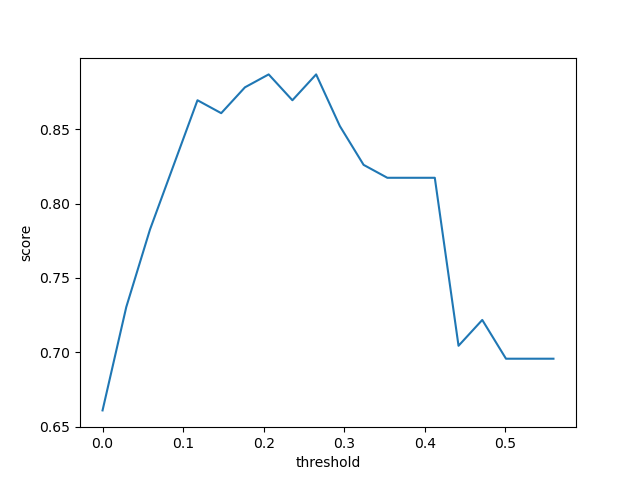


(A)


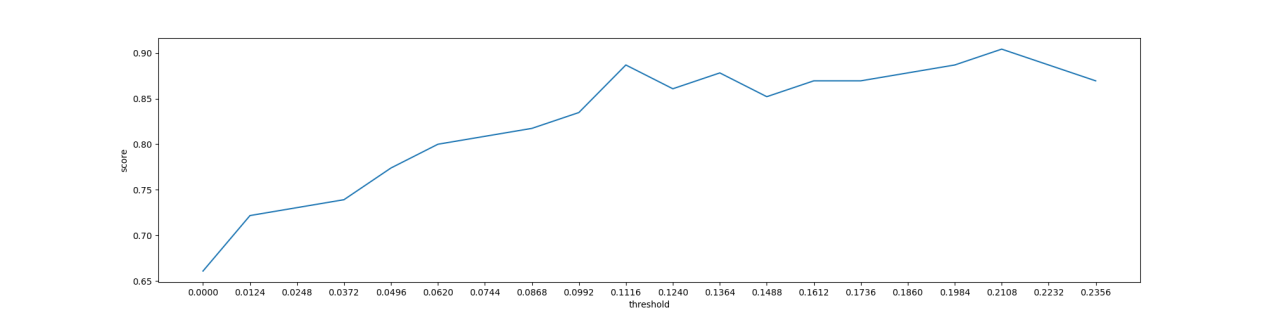


(B)

Fig.7 Radiomics features of PET image scaled using Scale algorithm and selected using the embedded capacity of logistic regression.(A) represent the relationship between the threshold of feature selection and the logistic regression model’s built by the features selected. (B) is the details of them.


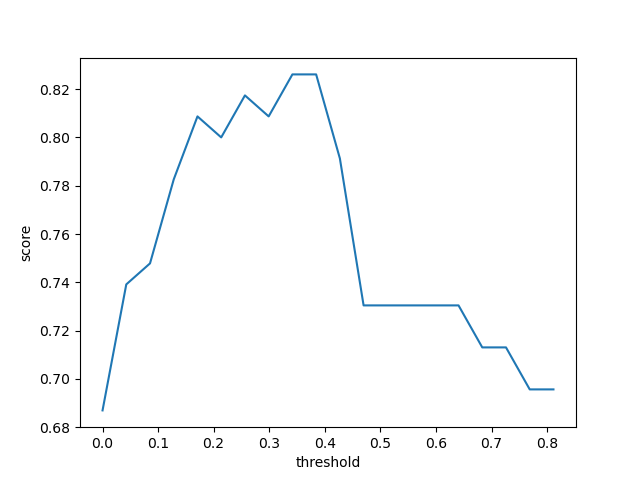


(A)


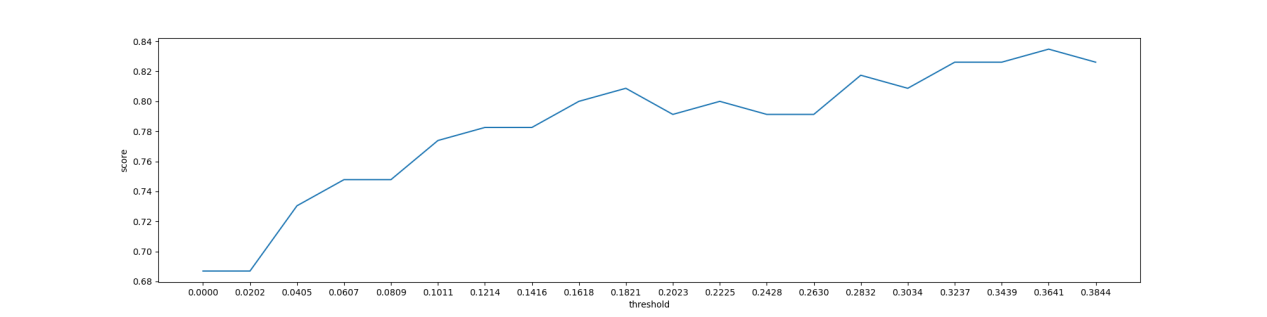


(B)

Fig.8 Radiomics features of PET image scaled using Scale algorithm without center -scaling and selected using the embedded capacity of logistic regression.(A) represent the relationship between the threshold of feature selection and the logistic regression model’s built by the features selected. (B) is the details of them.

**The results of the embedded capacity of decision tree**


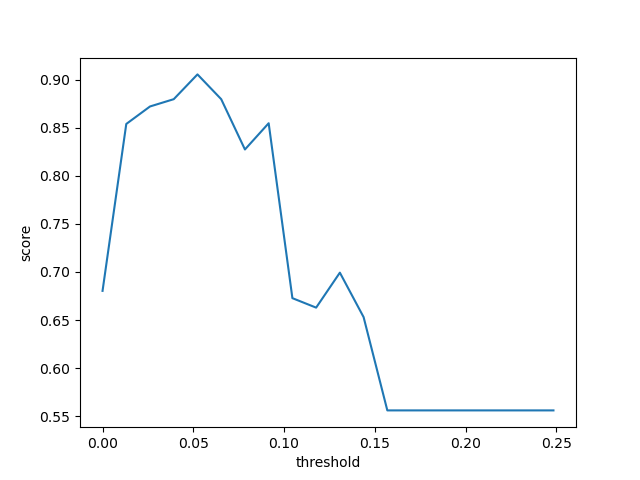


(A)


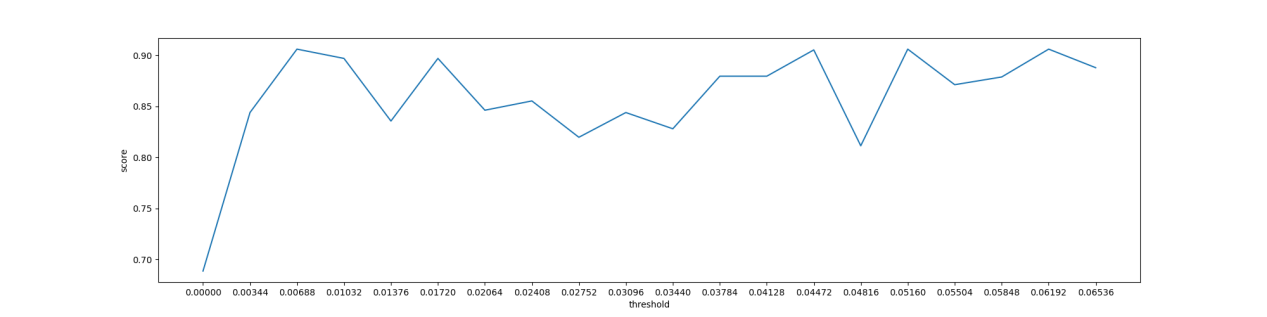


(B)

Fig.9 Radiomics features of CT image scaled using min-max algorithm and selected using the embedded capacity of decision tree.(A) represent the relationship between the threshold of feature selection and the logistic regression model’s built by the features selected. (B) is the details of them.


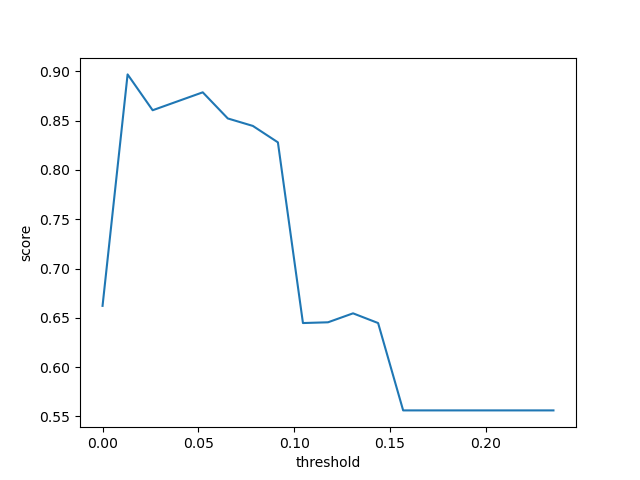


(A)


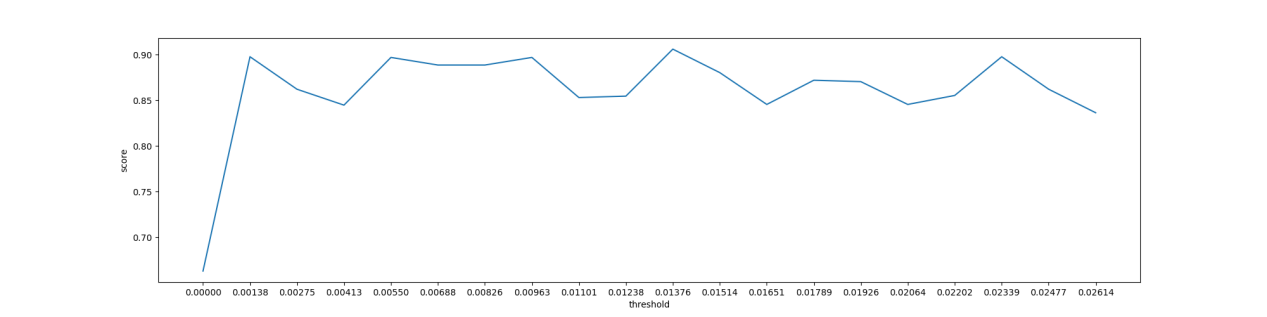


(B)

Fig.10 Radiomics features of CT image scaled using max-abs algorithm and selected using the embedded capacity of decision tree.(A) represent the relationship between the threshold of feature selection and the logistic regression model’s built by the features selected. (B) is the details of them.


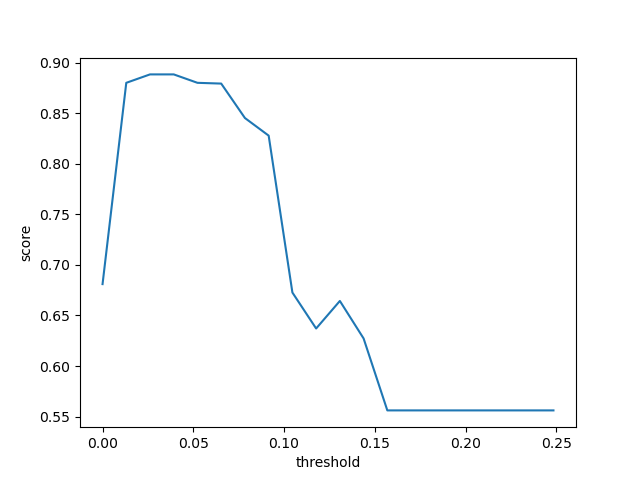


(A)


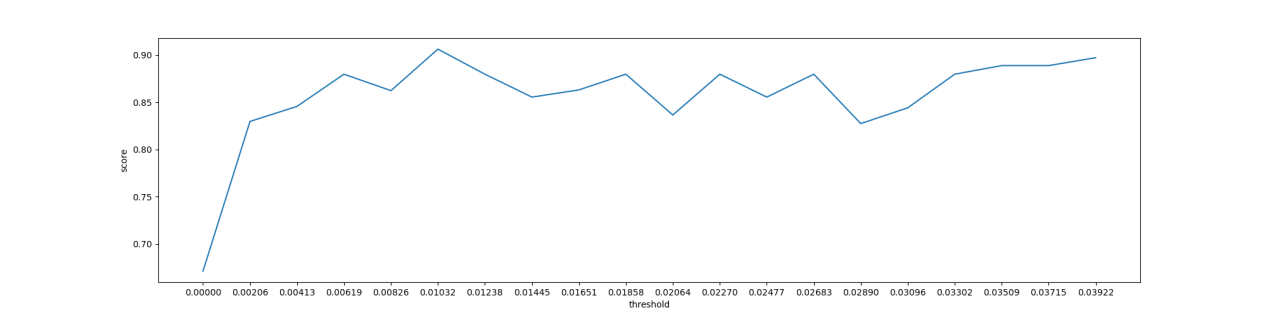


(B)

Fig.11 Radiomics features of CT image scaled using Scale algorithm and selected using the embedded capacity of decision tree.(A) represent the relationship between the threshold of feature selection and the logistic regression model’s built by the features selected. (B) is the details of them.


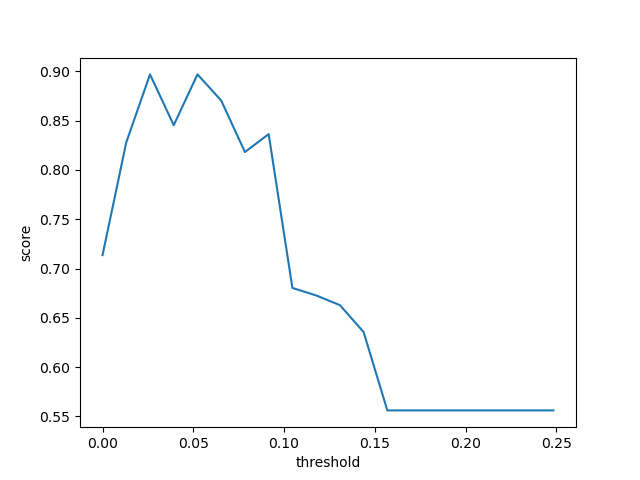


(A)


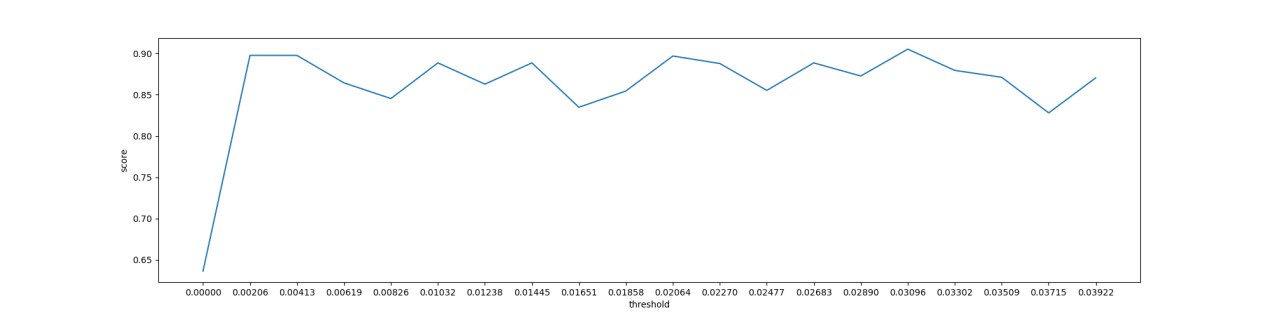


(B)

Fig.12 Radiomics features of CT image scaled using Scale algorithm without center -scaling and selected using the embedded capacity of decision tree.(A) represent the relationship between the threshold of feature selection and the logistic regression model’s built by the features selected. (B) is the details of them.


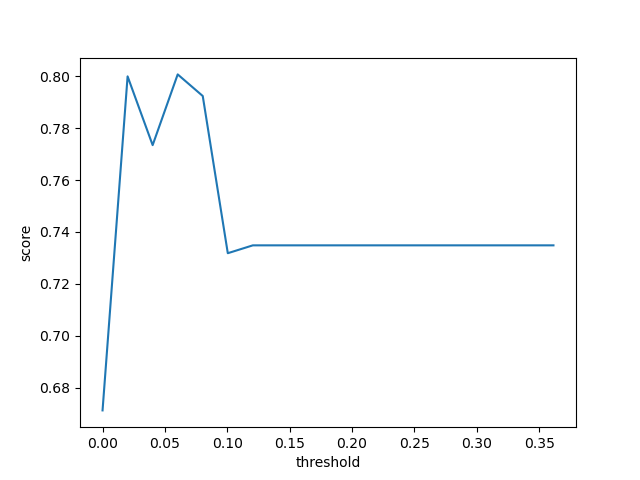


(A)


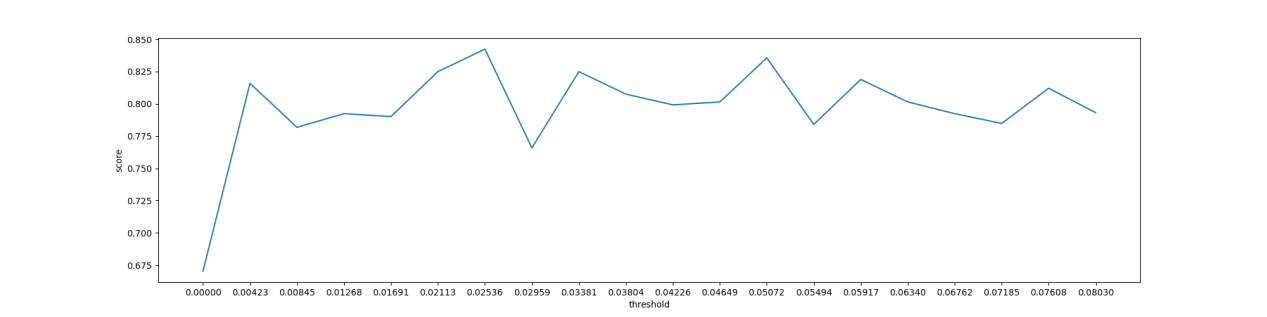


(B)

Fig.13 Radiomics features of PET image scaled using min-max algorithm and selected using the embedded capacity of decision tree.(A) represent the relationship between the threshold of feature selection and the logistic regression model’s built by the features selected. (B) is the details of them.


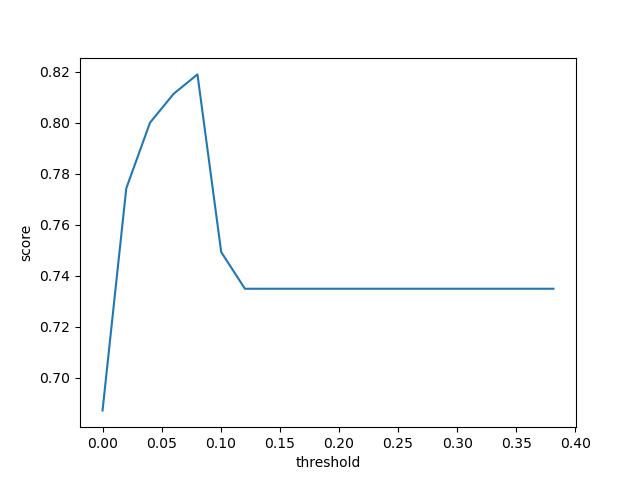


(A)


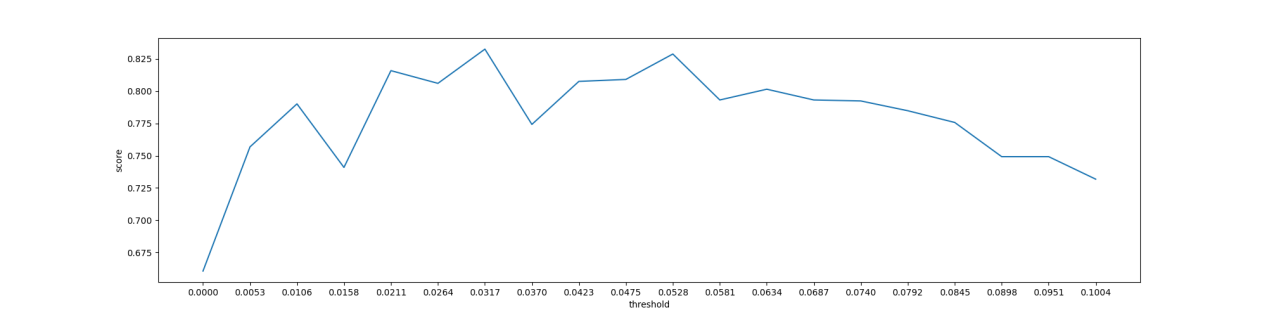


(B)

Fig.14 Radiomics features of PET image scaled using max-abs algorithm and selected using the embedded capacity of decision tree.(A) represent the relationship between the threshold of feature selection and the logistic regression model’s built by the features selected. (B) is the details of them.


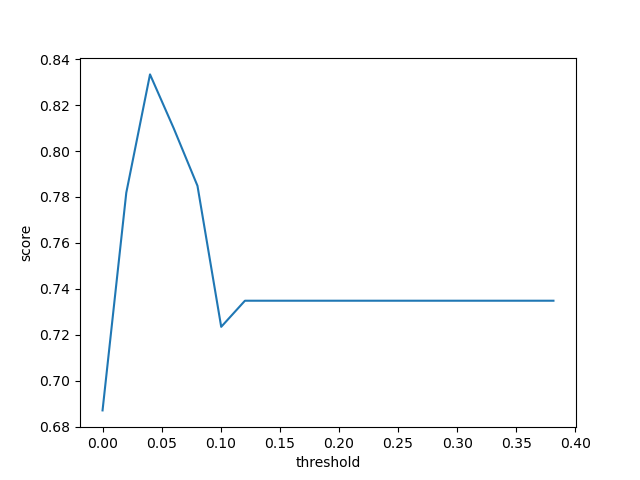


(A)


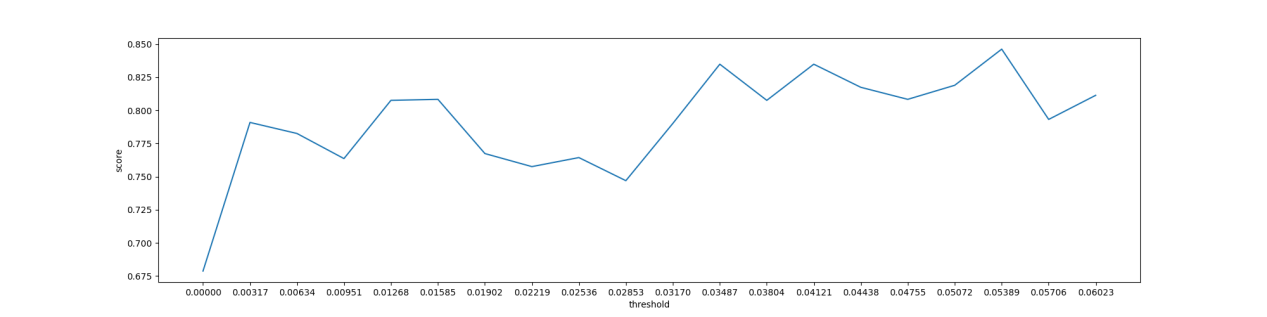


(B)

Fig.15 Radiomics features of PET image scaled using Scale algorithm and selected using the embedded capacity of decision tree.(A) represent the relationship between the threshold of feature selection and the logistic regression model’s built by the features selected. (B) is the details of them.


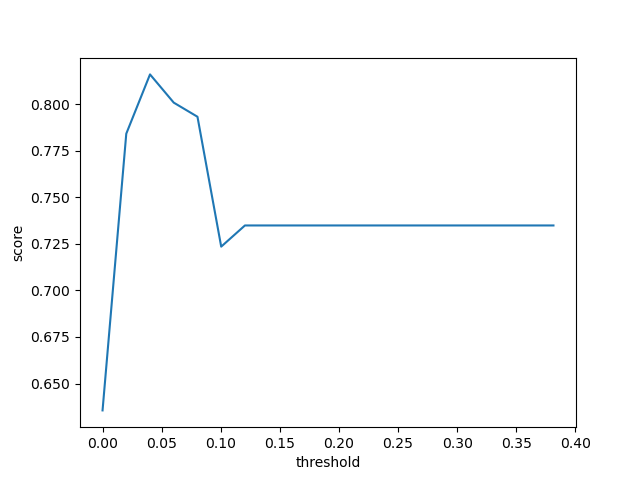


(A)


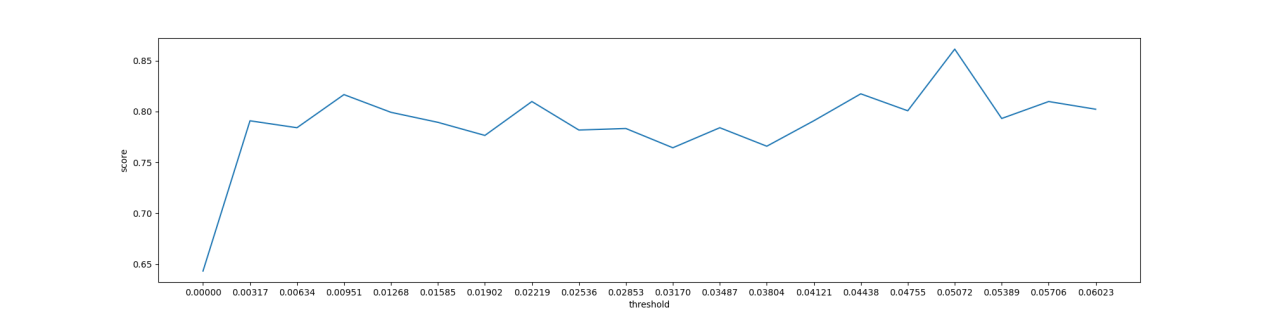


(B)

Fig.16 Radiomics features of PET image scaled using Scale algorithm without center -scaling and selected using the embedded capacity of decision tree.(A) represent the relationship between the threshold of feature selection and the logistic regression model’s built by the features selected. (B) is the details of them.

**The results of the embedded capacity of Random forest**

**
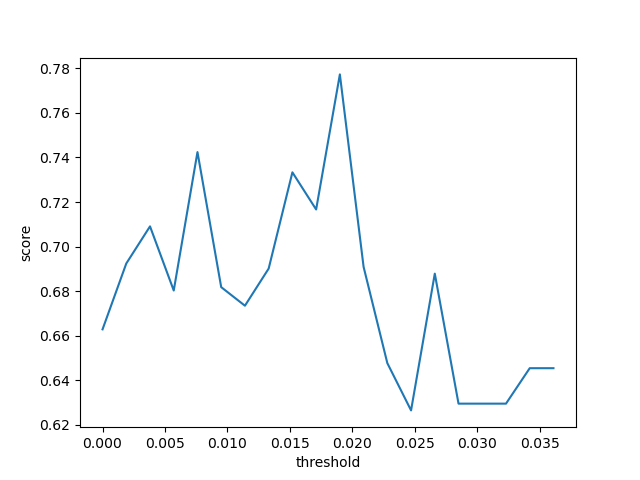
**

(A)

**
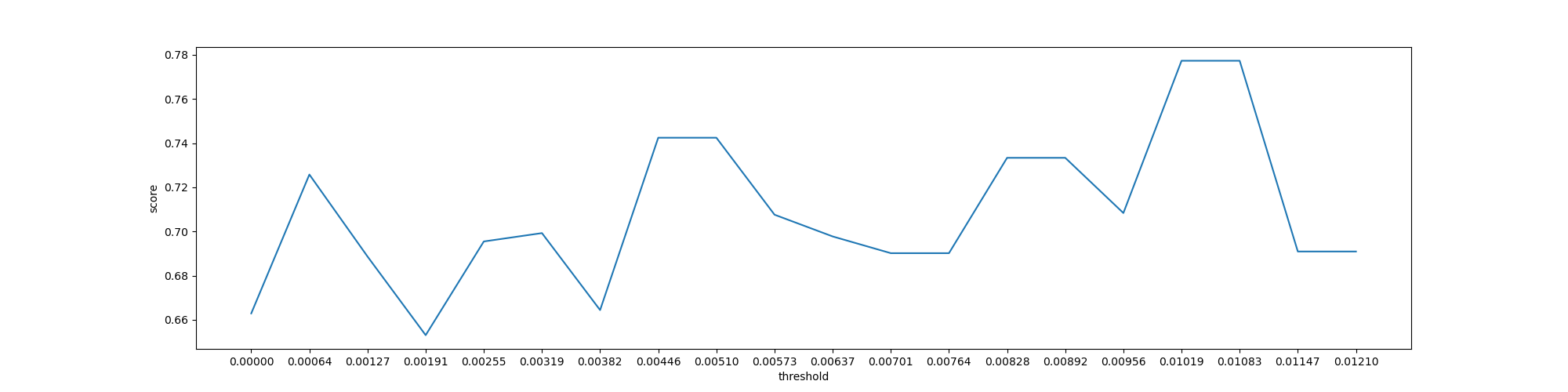
**

(B)

Fig.17 Radiomics features of CT image scaled using min-max algorithm and selected using the embedded capacity of random forest.(A) represent the relationship between the threshold of feature selection and the logistic regression model’s built by the features selected. (B) is the details of them.


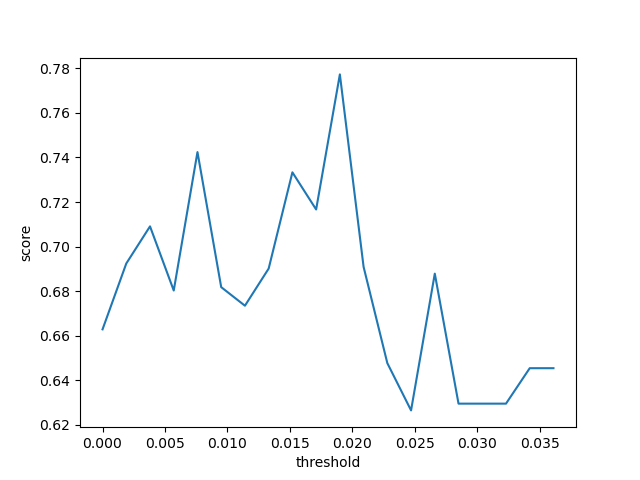


(A)


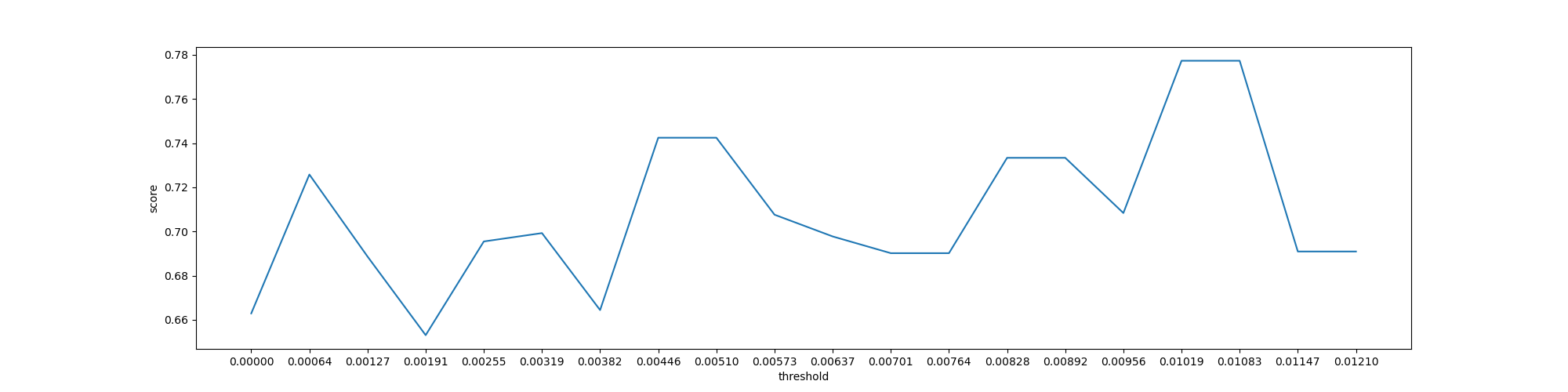


(B)

Fig.18 Radiomics features of CT image scaled using max-abs algorithm and selected using the embedded capacity of random forest.(A) represent the relationship between the threshold of feature selection and the logistic regression model’s built by the features selected. (B) is the details of them.


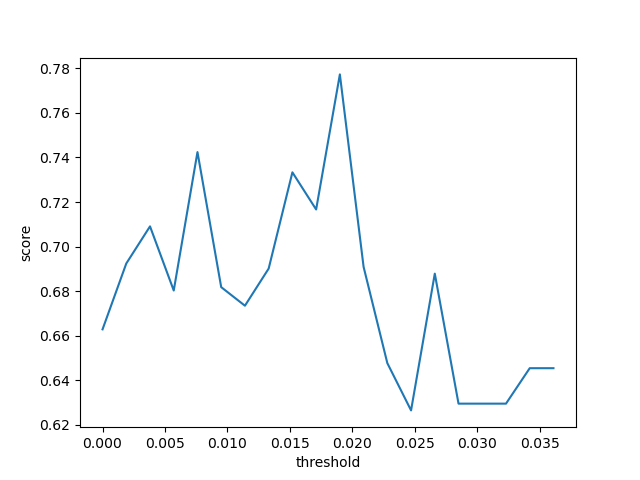


(A)


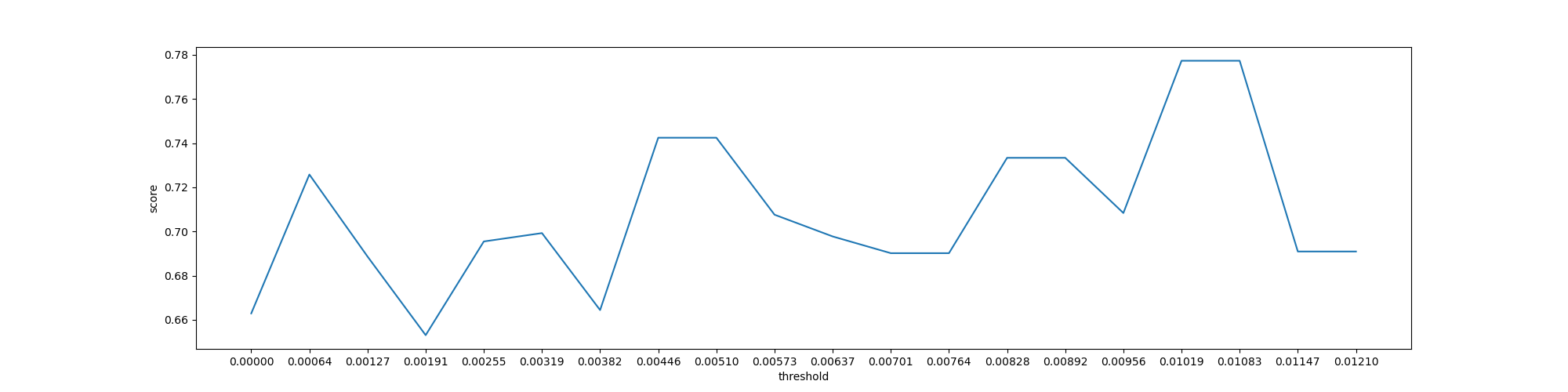


(B)

Fig.19 Radiomics features of CT image scaled using Scale algorithm and selected using the embedded capacity of random forest.(A) represent the relationship between the threshold of feature selection and the logistic regression model’s built by the features selected. (B) is the details of them.


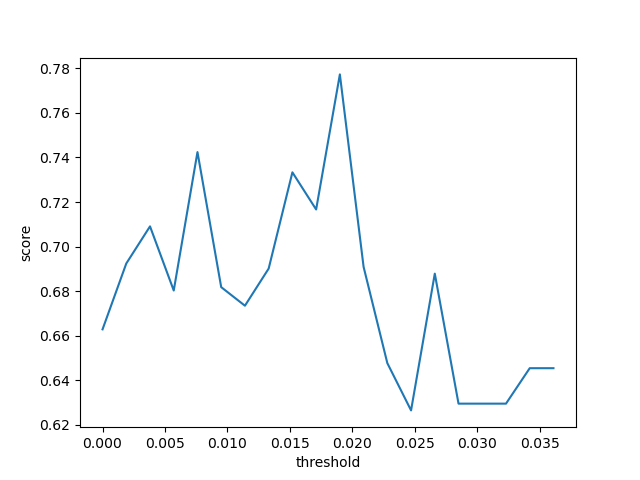


(A)


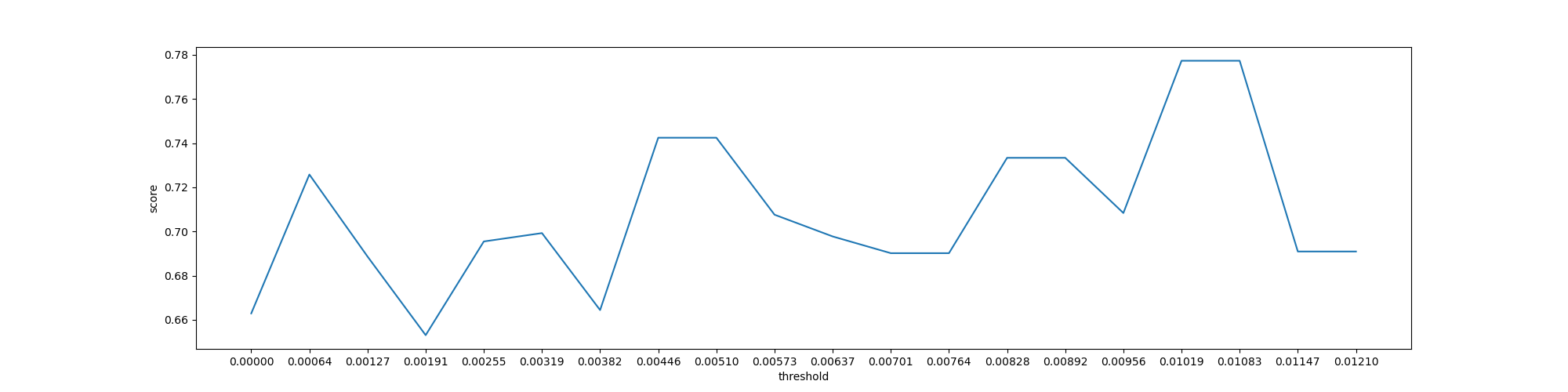


(B)

Fig.20 Radiomics features of CT image scaled using Scale algorithm without center -scaling and selected using the embedded capacity of random forest.(A) represent the relationship between the threshold of feature selection and the logistic regression model’s built by the features selected. (B) is the details of them.


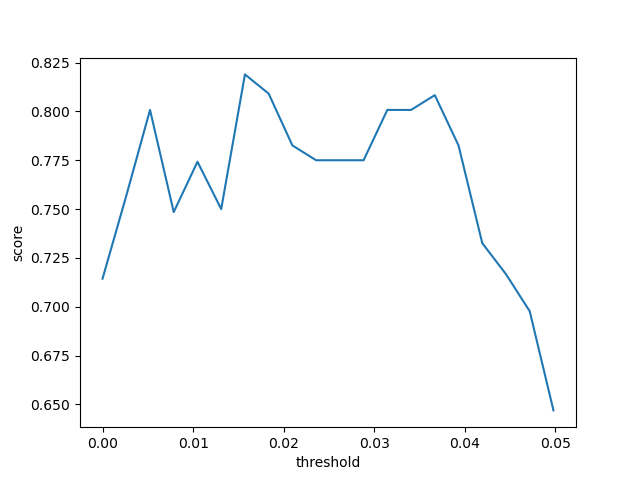


(A)


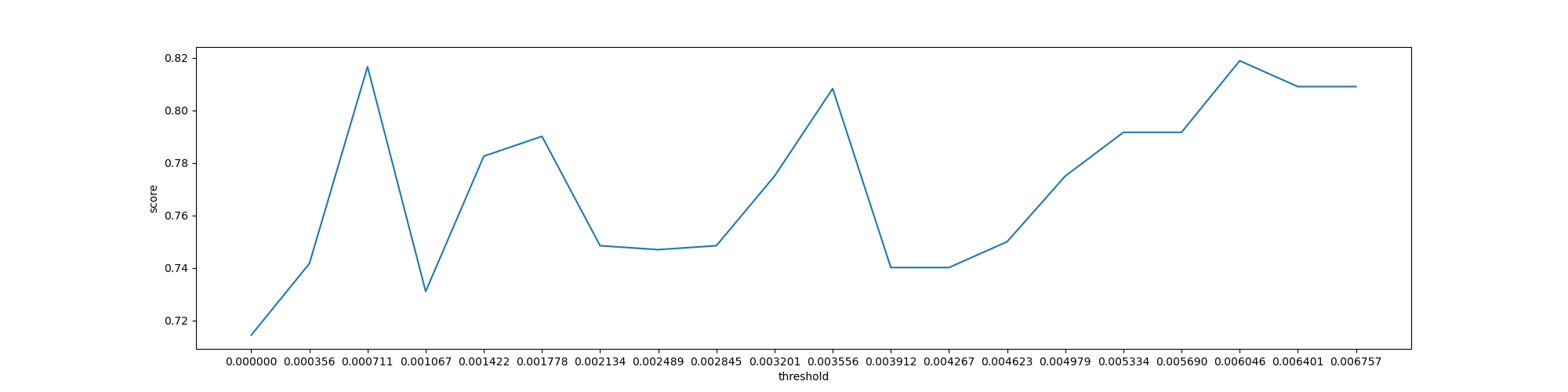


(B)

Fig.21 Radiomics features of PET image scaled using min-max algorithm and selected using the embedded capacity of random forest.(A) represent the relationship between the threshold of feature selection and the logistic regression model’s built by the features selected. (B) is the details of them.


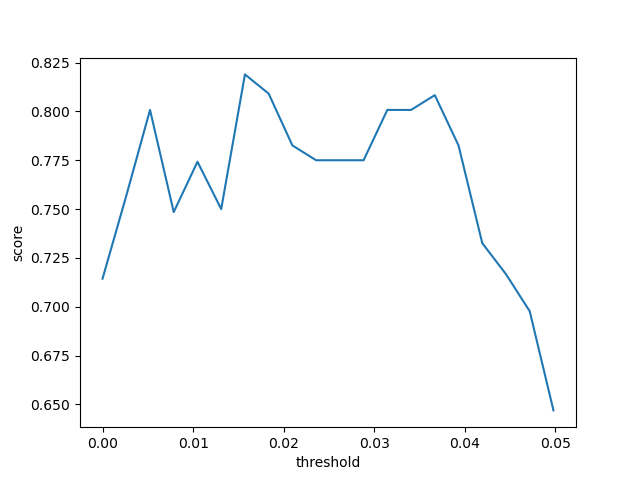


(A)


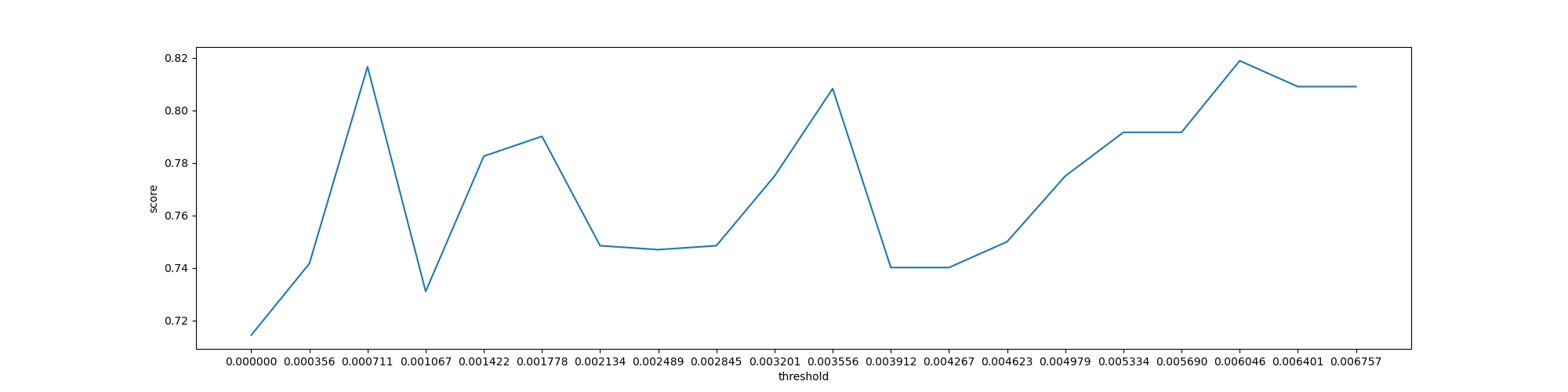


(B)

Fig.22 Radiomics features of PET image scaled using max-abs algorithm and selected using the embedded capacity of random forest.(A) represent the relationship between the threshold of feature selection and the logistic regression model’s built by the features selected. (B) is the details of them.


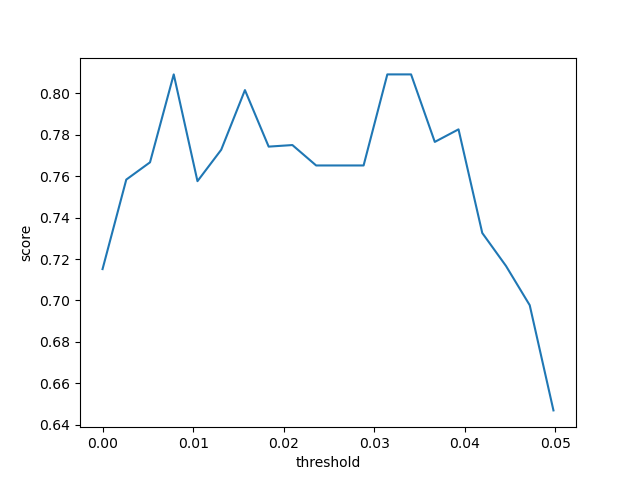


(A)


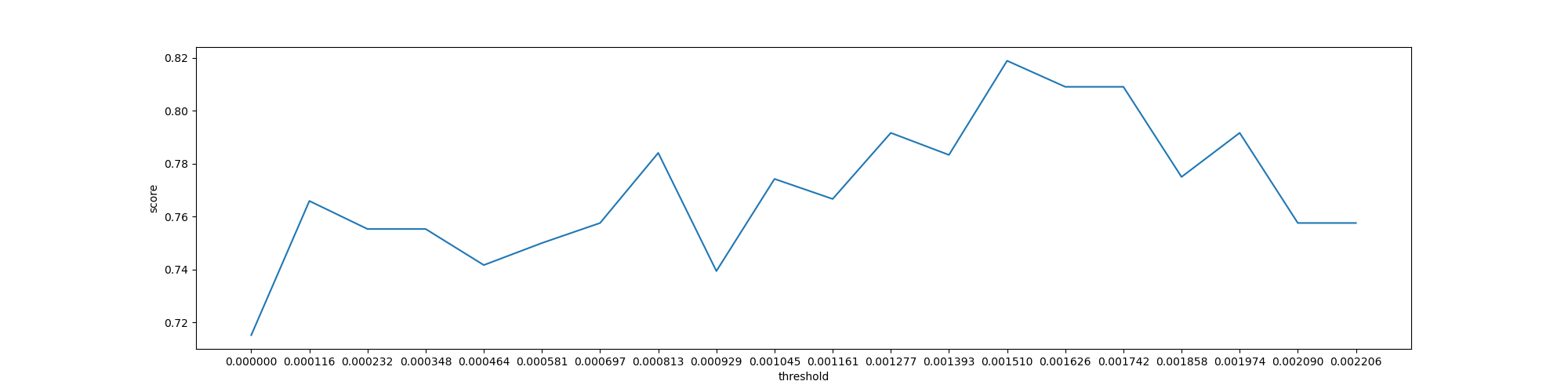


(B)

Fig.23 Radiomics features of PET image scaled using Scale algorithm and selected using the embedded capacity of random forest.(A) represent the relationship between the threshold of feature selection and the logistic regression model’s built by the features selected. (B) is the details of them.


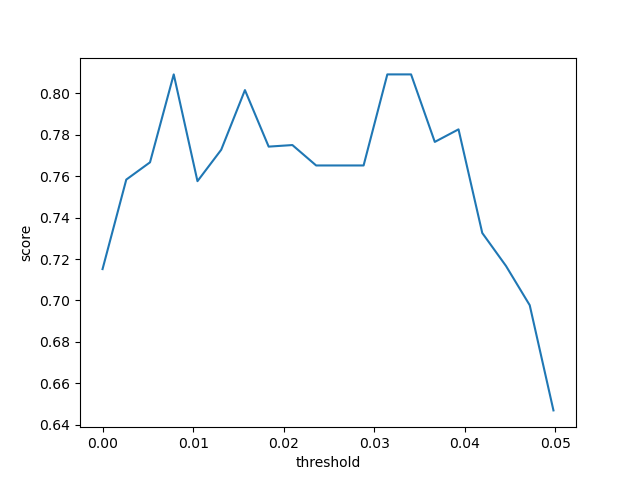


(A)


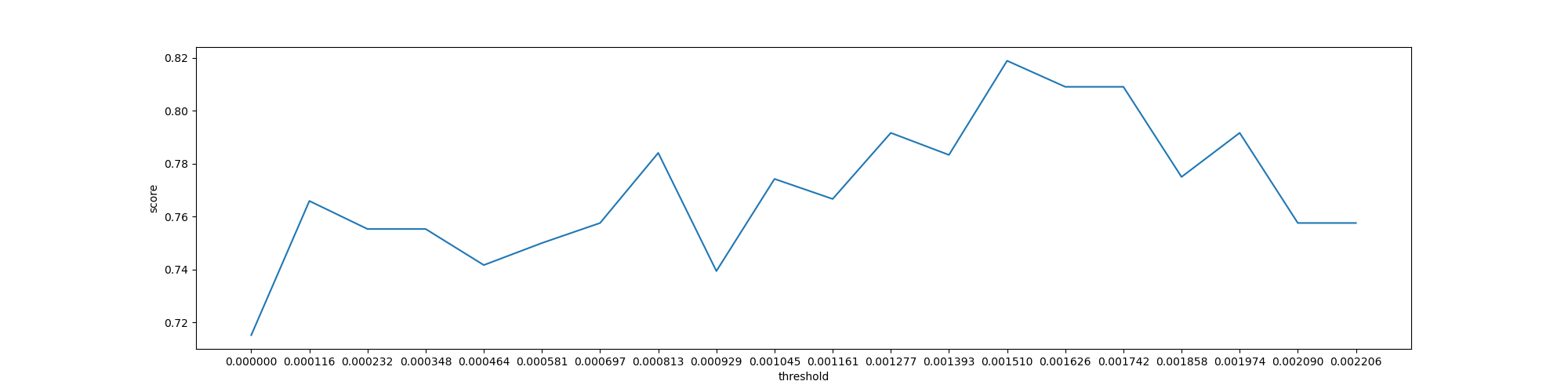


(B)

Fig.24 Radiomics features of PET image scaled using Scale algorithm without center -scaling and selected using the embedded capacity of random forest.(A) represent the relationship between the threshold of feature selection and the logistic regression model’s built by the features selected. (B) is the details of them.
