# Supplementary material for: Applications of radiomics-based analysis pipeline for predicting epidermal growth factor receptor mutation status
Source: Biomed Eng Online. 2023 Feb 21;22:17. doi: 10.1186/s12938-022-01049-9 (PMC9945395; doi:10.1186/s12938-022-01049-9)
Supplement: Supplementary file 2 — Additional file 2. Additional Results. [file 12938_2022_1049_MOESM2_ESM.docx]

Additional Results

**The results of feature selection**

Results of radiomic feature selection are shown in the Additional Table Contents[see Additional file 3 and Additional file 4] .In the tables,0 means the features are removed and 1 means the features are selected.

**The results of Variance threshold**

Results of radiomics features of CT images selected using Variance threshold are shown in the Supplementary Table Contents[see Additional file 5] .The radiomics features of PET images selected results using Variance threshold are shown in the Supplementary Table Contents[see Additional file 6] .

The variance of radiomics features and the results of features selected using Variance threshold are shown in these tables.

**The results of t-test**

Results of radiomics features of CT images selected by t-test are shown in the Supplementary Table Contents[see Additional file 7-10] .The radiomics features of PET images selected results using t-test are shown in the Supplementary Table Contents[see Additional file 11-14] .

The results of Levene test and t-test test of radiomics features are shown in these tables. The results of features selected using t-test are also shown in these tables.

**The results of Mutual information**

Results of radiomics features of CT images selected by mutual information are shown in the Additional Table Contents[see Additional file 15-18] . The radiomics features of PET images selected results using t-test are shown in the Supplementary Table Contents[see Additional file 19-22] .

The results of mutual information of Radiomics features and the results of features selected using mutual information are shown in these tables.

**The results of embedded solutions**

**The results of the embedded capacity of logistic regression**


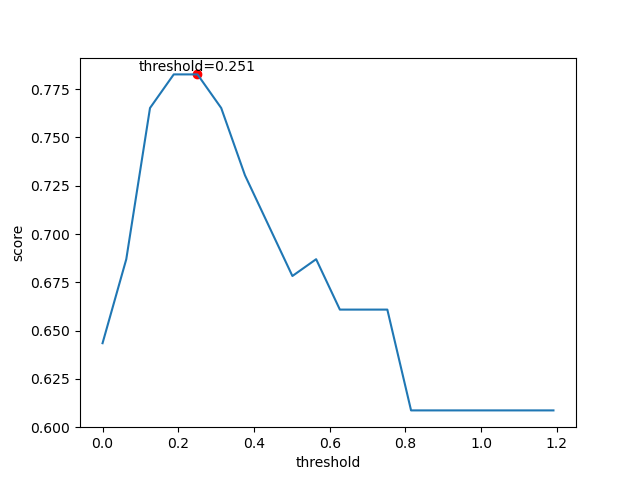
Fig.1 In CT images, Radiomics features scaled by min-max algorithm and selected by the embedded capacity of logistic regression. Threshold: the threshold of feature selection. Score: the score of the logistic regression model built by the selected features.


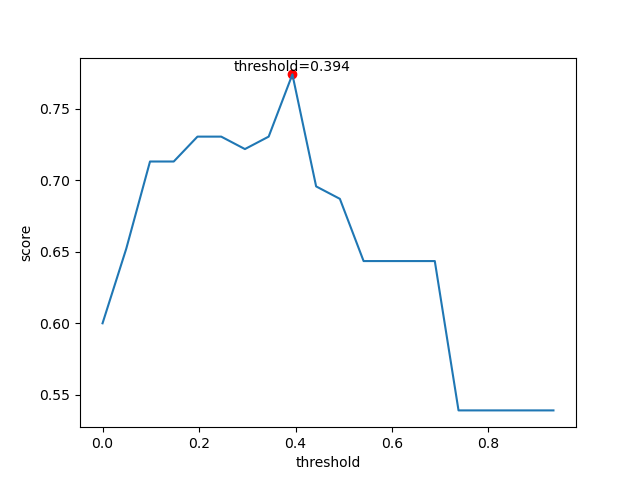
Fig.2 In CT images, Radiomics features scaled by max-abs algorithm and selected by the embedded capacity of logistic regression. Threshold: the threshold of feature selection. Score: the score of the logistic regression model built by the selected features.


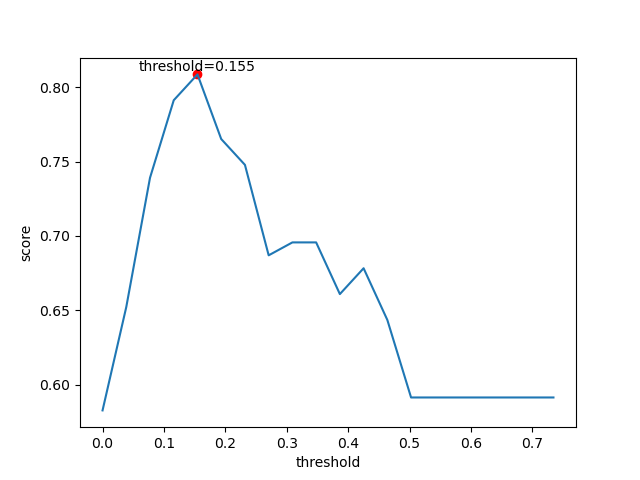
Fig.3 In CT images, Radiomics features scaled by Scale algorithm and selected by the embedded capacity of logistic regression. Threshold: the threshold of feature selection. Score: the score of the logistic regression model built by the selected features.


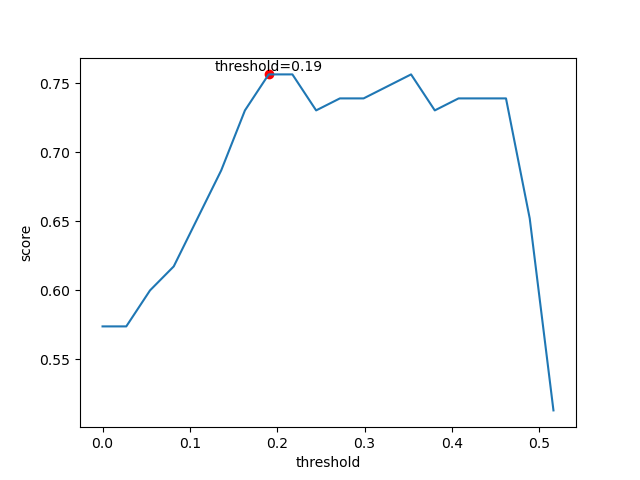
Fig.4 In CT images, Radiomics features scaled by Scale algorithm without center -scaling and selected by the embedded capacity of logistic regression. Threshold: the threshold of feature selection. Score: the score of the logistic regression model built by the selected features.


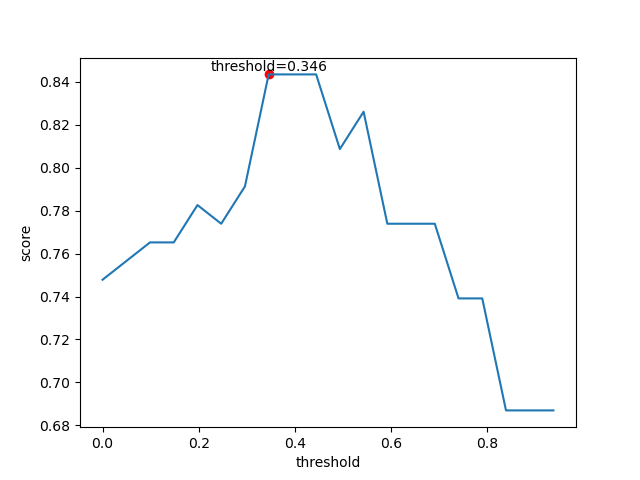
Fig.5 In PET images, Radiomics features scaled by min-max algorithm and selected by the embedded capacity of logistic regression. Threshold: the threshold of feature selection. Score: the score of the logistic regression model built by the selected features.


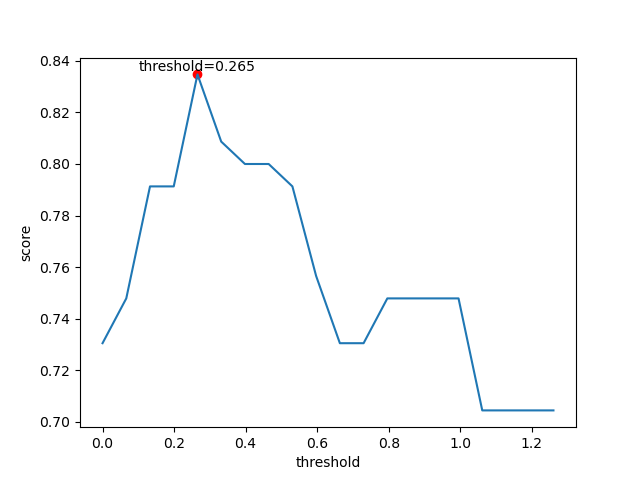
Fig.6 In PET images, Radiomics features scaled by max-abs algorithm and selected by the embedded capacity of logistic regression. Threshold: the threshold of feature selection. Score: the score of the logistic regression model built by the selected features.


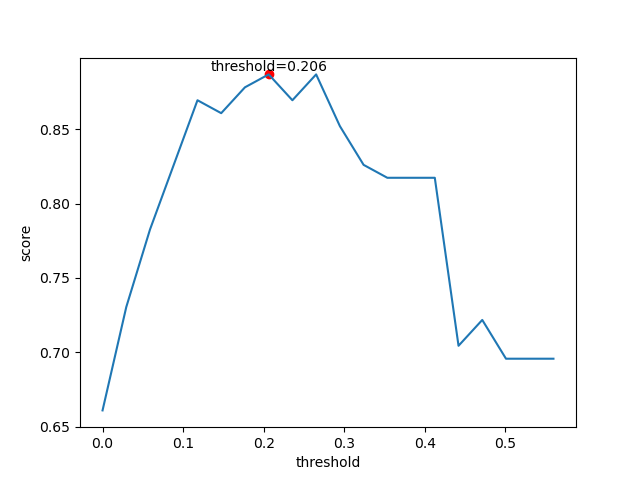
Fig.7 In PET images, Radiomics features scaled by Scale algorithm and selected by the embedded capacity of logistic regression. Threshold: the threshold of feature selection. Score: the score of the logistic regression model built by the selected features.


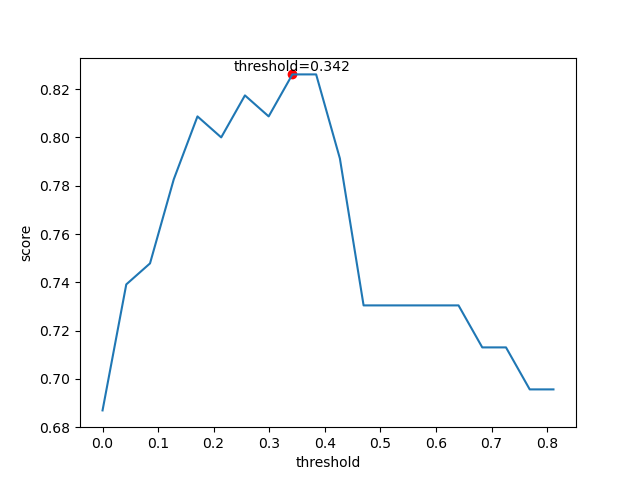
Fig.8 In PET images, Radiomics features scaled by Scale algorithm without center -scaling and selected by the embedded capacity of logistic regression. Threshold: the threshold of feature selection. Score: the score of the logistic regression model built by the selected features.

**The results of the embedded capacity of decision tree**


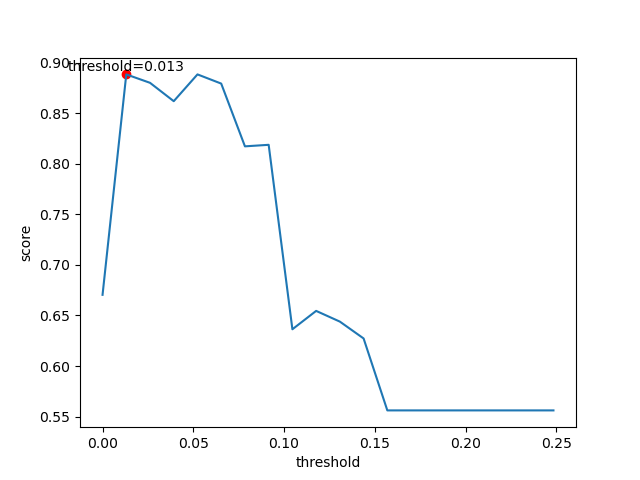
Fig.9 In CT images, Radiomics features scaled by min-max algorithm and selected by the embedded capacity of decision tree. Threshold: the threshold of feature selection. Score: the score of the decision tree model built by the selected features.


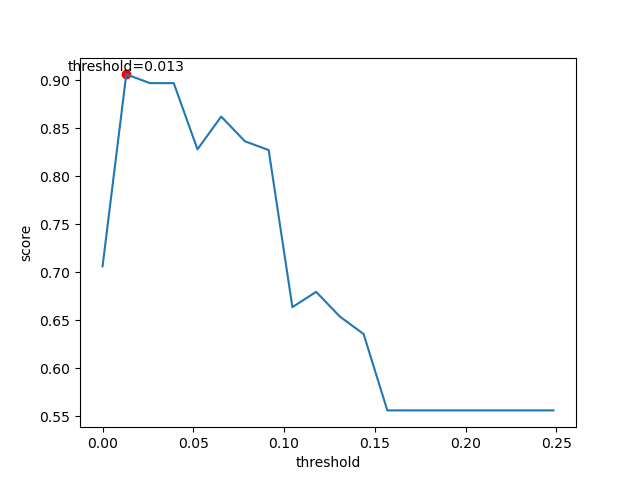
Fig.10 In CT images, Radiomics features scaled by max-abs algorithm and selected by the embedded capacity of decision tree. Threshold: the threshold of feature selection.Score: the score of the decision tree model built by the selected features.


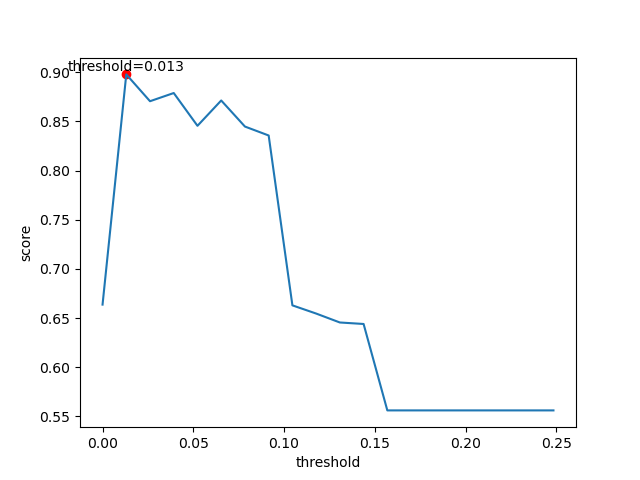
Fig.11 In CT images, Radiomics features scaled by Scale algorithm and selected by the embedded capacity of decision tree. Threshold: the threshold of feature selection.Score: the score of the decision tree model built by the selected features.


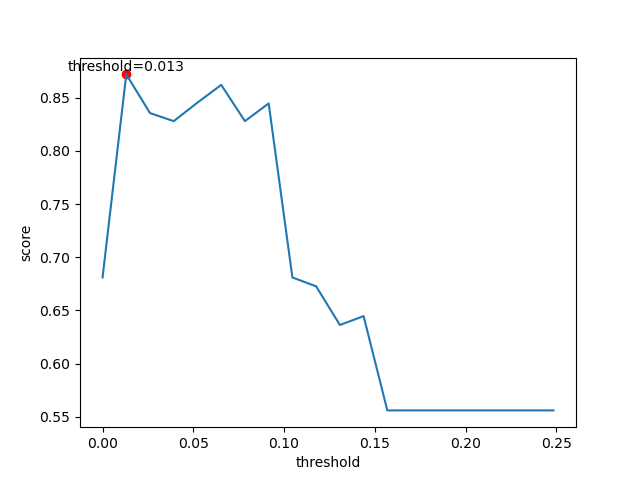
Fig.12 In CT images, Radiomics features scaled by Scale algorithm without center -scaling and selected by the embedded capacity of decision tree. Threshold: the threshold of feature selection. Score: the score of the decision tree model built by the selected features.


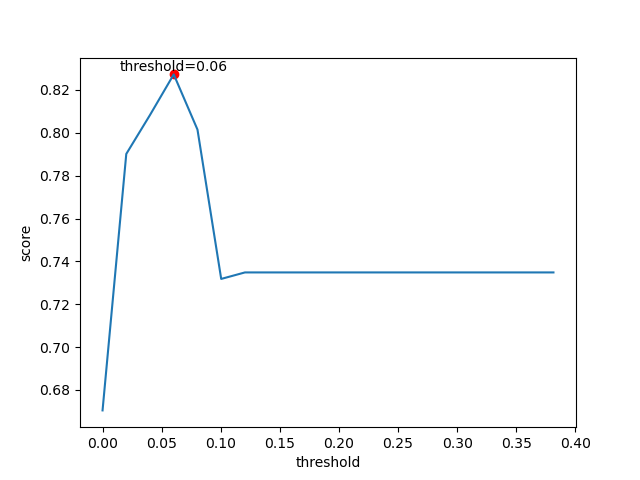
Fig.13 In PET images, Radiomics features scaled by min-max algorithm and selected by the embedded capacity of decision tree. Threshold: the threshold of feature selection. Score: the score of the decision tree model built by the selected features.


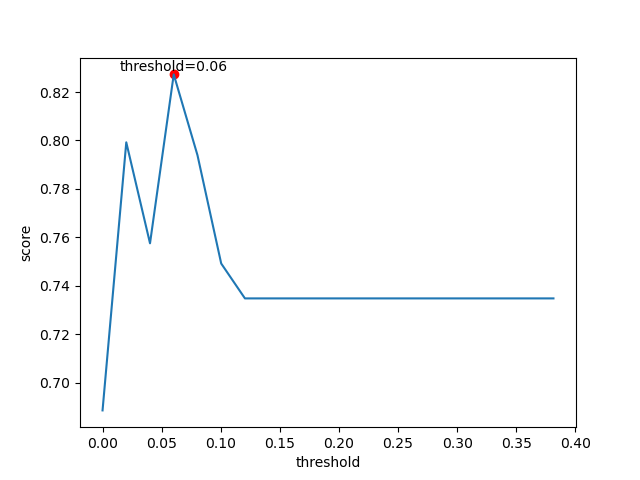
Fig.14 In PET images, Radiomics features scaled by max-abs algorithm and selected by the embedded capacity of decision tree. Threshold: the threshold of feature selection. Score: the score of the decision tree model built by the selected features.


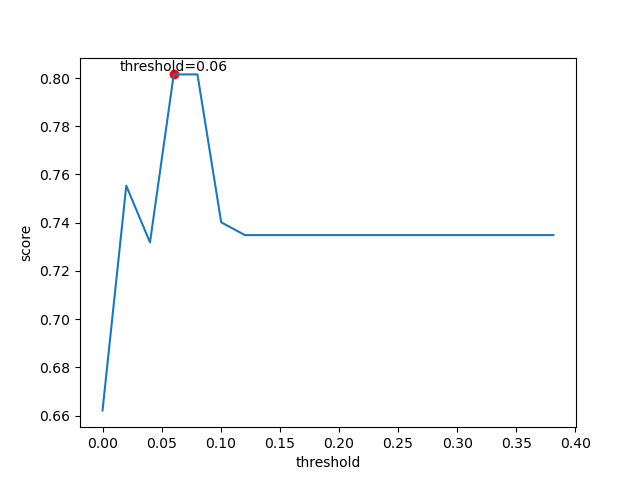
Fig.15 In PET images, Radiomics features scaled by Scale algorithm and selected by the embedded capacity of decision tree. Threshold: the threshold of feature selection. Score: the score of the decision tree model built by the selected features.


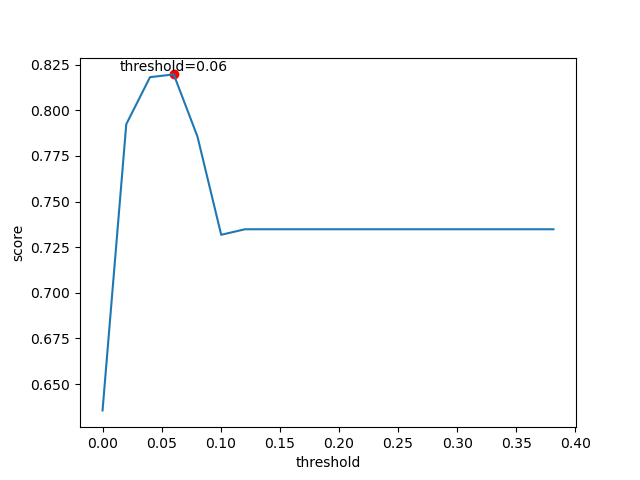
Fig.16 In PET images, Radiomics features scaled by Scale algorithm without center -scaling and selected by the embedded capacity of decision tree. Threshold: the threshold of feature selection. Score: the score of the decision tree model built by the selected features.

**The results of the embedded capacity of Random forest**


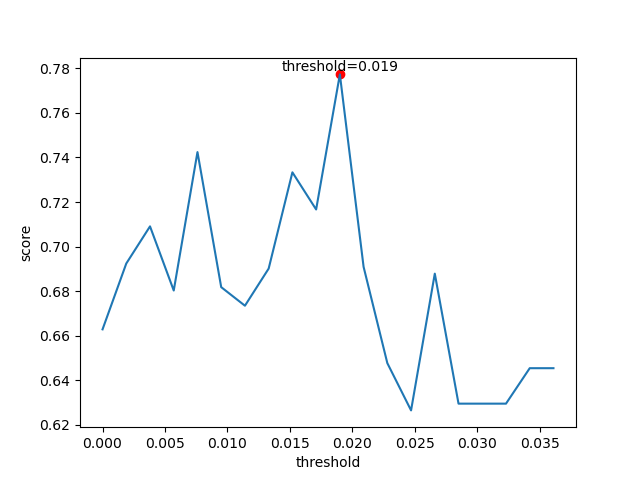
Fig.17 In CT images, Radiomics features scaled by min-max algorithm and selected by the embedded capacity of random forest. Threshold: the threshold of feature selection. Score: the score of the random forest model built by the selected features.


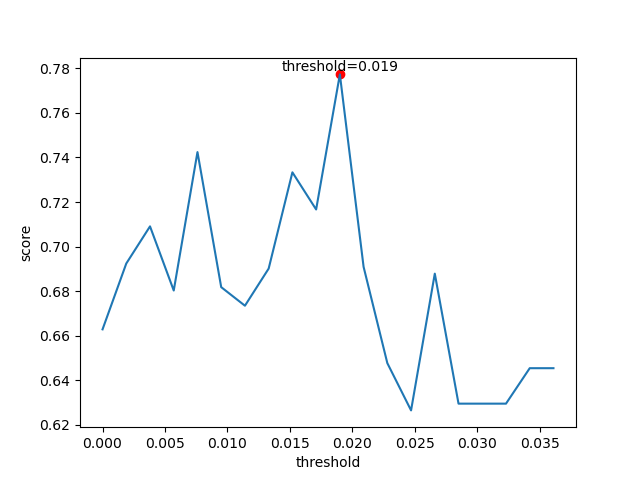


Fig.18 In CT images, Radiomics features scaled by max-abs algorithm and selected by the embedded capacity of random forest. Threshold: the threshold of feature selection. Score: the score of the random forest model built by the selected features.


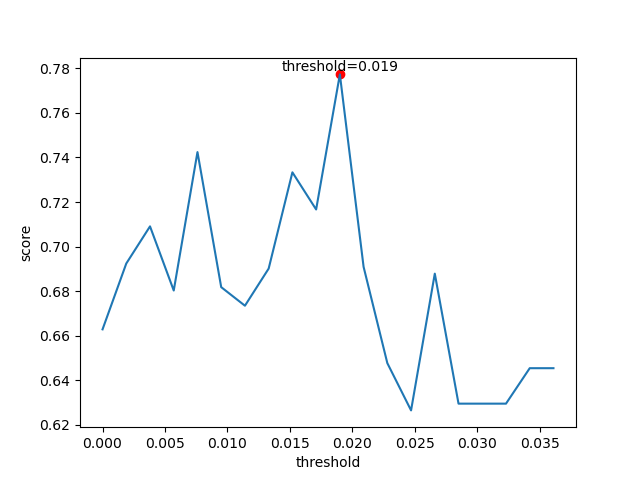
Fig.19 In CT images, Radiomics features scaled by Scale algorithm and selected by the embedded capacity of random forest. Threshold: the threshold of feature selection. Score: the score of the random forest model built by the selected features.


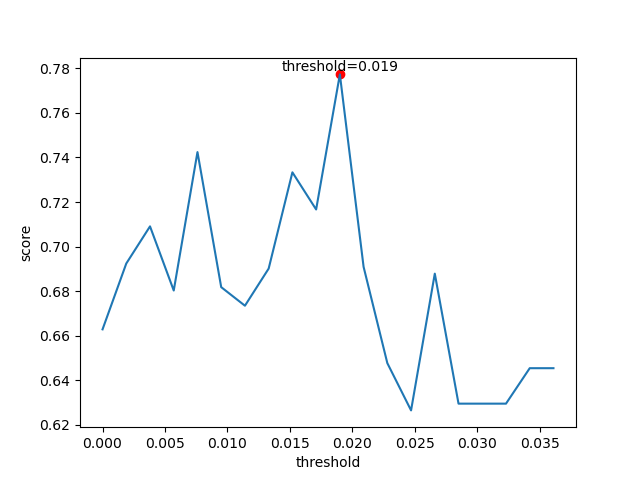
Fig.20 In CT images, Radiomics features scaled by Scale algorithm without center -scaling and selected by the embedded capacity of random forest. Threshold: the threshold of feature selection. Score: the score of the random forest model built by the selected features.


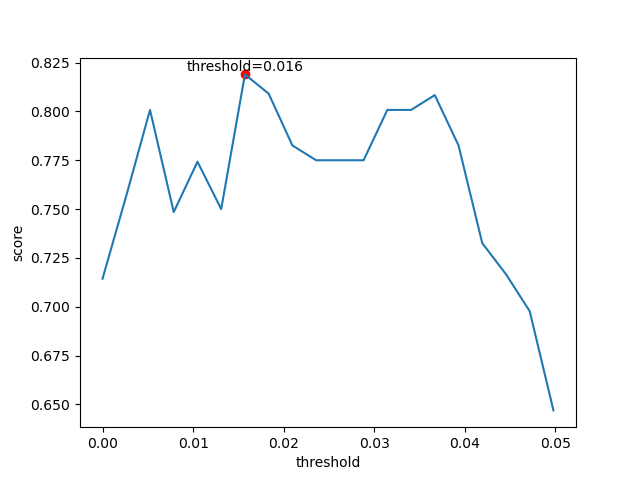
Fig.21 In PET images, Radiomics features scaled by min-max algorithm and selected by the embedded capacity of random forest. Threshold: the threshold of feature selection. Score: the score of the random forest model built by the selected features.


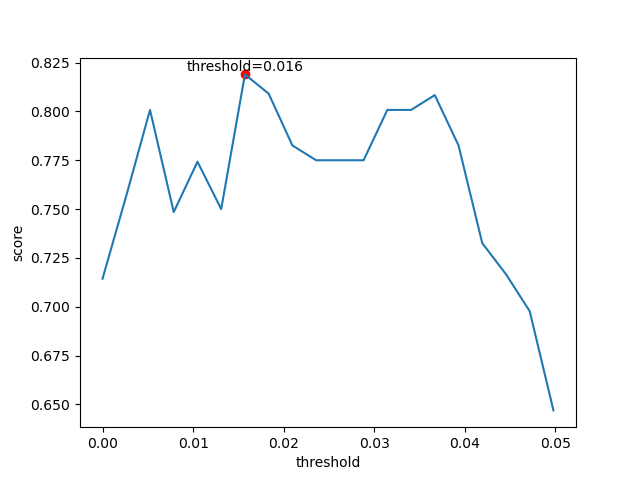
Fig.22 In PET images, Radiomics features scaled by max-abs algorithm and selected by the embedded capacity of random forest. Threshold: the threshold of feature selection. Score: the score of the random forest model built by the selected features.


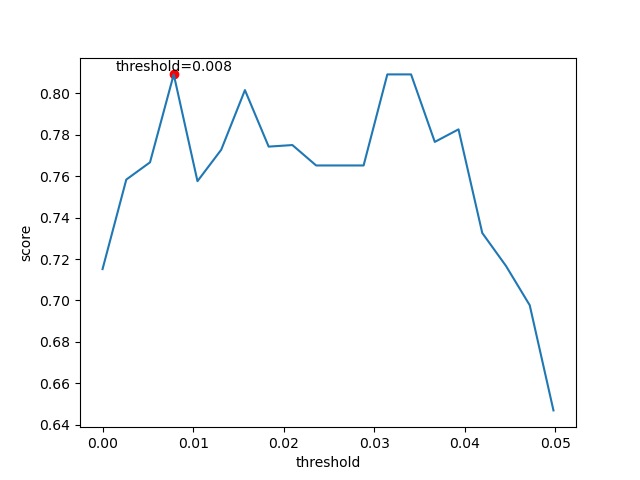
Fig.23 In PET images, Radiomics features scaled by Scale algorithm and selected by the embedded capacity of random forest. Threshold: the threshold of feature selection. Score: the score of the random forest model built by the selected features.


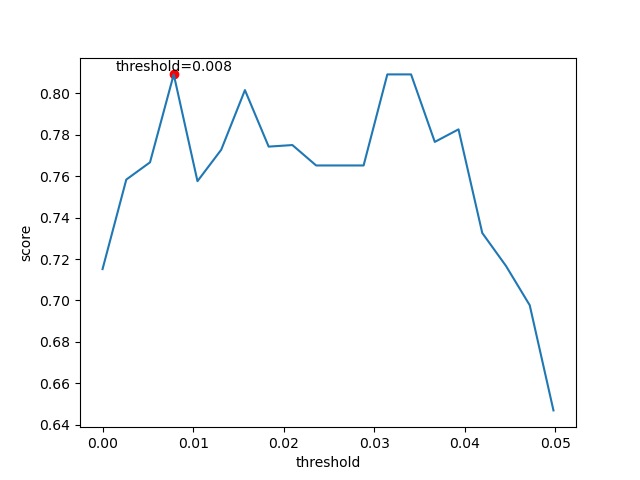
Fig.24 In PET images, Radiomics features scaled by Scale algorithm without center -scaling and selected by the embedded capacity of random forest. Threshold: the threshold of feature selection. Score: the score of the random forest model built by the selected features.
